# Maternal consumption of a fermented diet protects offspring against intestinal inflammation by regulating the gut microbiota

**DOI:** 10.1080/19490976.2022.2057779

**Published:** 2022-05-04

**Authors:** Cheng Wang, Siyu Wei, Bojing Liu, Fengqin Wang, Zeqing Lu, Mingliang Jin, Yizhen Wang

**Affiliations:** National Engineering Laboratory for Feed Safety and Pollution Prevention and Controlling; Key Laboratory of Molecular Animal Nutrition, Ministry of Education; Key Laboratory of Animal Nutrition and Feed Science (Eastern of China), Ministry of Agriculture and Rural Affairs; Key Laboratory of Animal Feed and Nutrition of Zhejiang Province; Institute of Feed Science, Zhejiang University, 866 Yuhang Tang Road, Hangzhou, 310058, PR China

**Keywords:** Intestinal inflammation, maternal fermented diet, neonatal gut microbiota, sows and piglets, gut microbiome, milk metabolome

## Abstract

The neonatal intestinal tract is immature and can be easily infected by pathogens causing inflammation. Maternal diet manipulation is a promising nutritional strategy to enhance the gut health of offspring. A fermented diet is a gut microbiota targeting diet containing live probiotics and their metabolites, which benefit the gut and overall health host. However, it remains unclear how a maternal fermented diet (MFD) affects neonatal intestinal inflammation. Here, *in vivo* and *in vitro* models together with multi-omics analysis were applied to investigate the impacts and the underlying mechanism through which an MFD prevents from gut inflammation in neonates. An MFD remarkably improved the performance of both sows and piglets and significantly altered the gut microbiome and milk metabolome of sows. In addition, the MFD significantly accelerated the maturation of the gut microbiota of neonates and increased the abundance of gut *Lactobacillus* and the microbial functions of amino acid-related enzymes and glucose metabolism on the weaning day. Notably, the MFD reduced susceptibility to colonic inflammation in offspring. The fecal microbiota of sows was then transplanted into mouse dams and it was found that the mouse dams and pups in the MFD group alleviated the LPS-induced decrease in gut *Lactobacillus* abundance and barrier injury. Milk L-glutamine (GLN) and gut *Lactobacillus reuteri* (LR) were found as two of the main MFD-induced sow effectors that contributed to the gut health of piglets. The properties of LR and GLN in modulating gut microbiota and alleviating colonic inflammation by inhibiting the phosphorylation of p38 and JNK and activation of Caspase 3 were further verified. These findings provide the first data revealing that an MFD drives neonate gut microbiota development and ameliorates the colonic inflammation by regulating the gut microbiota. This fundamental evidence might provide references for modulating maternal nutrition to enhance early-life gut health and prevent gut inflammation.

## Graphical abstract

**Figure:**
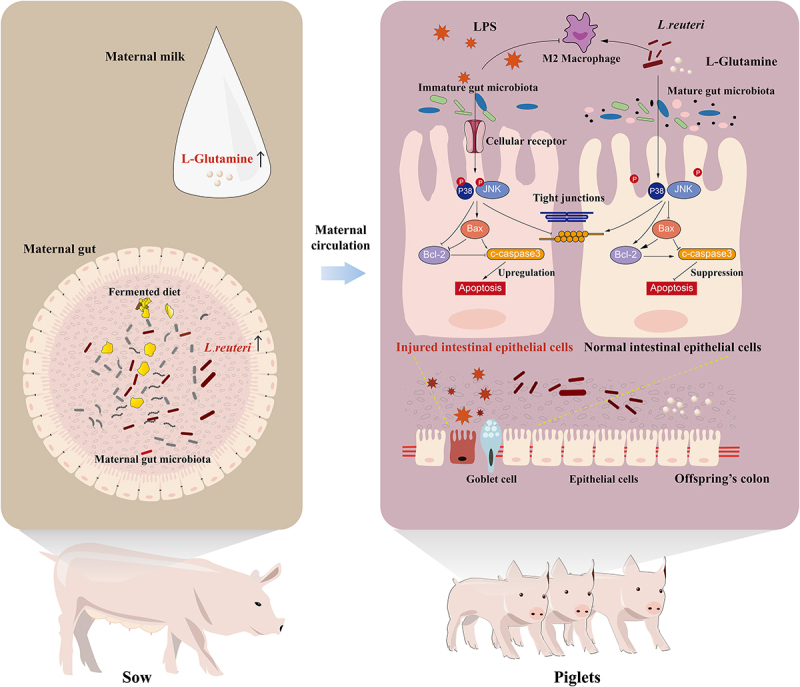

## Introduction

Early life is a critical period in which the gut microbiota, the physical barrier functions of the intestine, and the immune system are under development in infants. There is increasing evidence that the maturation and health of the neonatal gut may play an important role in programming health and disease later in life.^1^ Disturbances in early-life gut development have been found to be associated with the occurrence of inflammatory gut injury in both children and adults.^2–4^ Maternal diet can affect early postnatal gut development and health primarily through milk and gut microbiota.^5,6^ Maternal milk affects the maturation of the gut in infants by supplying basic nutrients and bioactive components such as immunoglobulin, oligosaccharides, short-chain fatty acids, antimicrobial peptides, and microbes.^7^ The maternal gut microbiota shapes the early-life gut microbiota of the offspring through the entero-mammary axis^8^ and drives early postnatal innate immune development and metabolic phenotypes.^9,10^

Dietary variables can dramatically and rapidly affect the gut microbiome.^11^ Fermented foods, such as kombucha, yogurt, and kimchi, constitute one type of gut microbiota–targeting diet, containing not only foundational nutrients (carbohydrates, proteins, and fats) but also probiotics, prebiotics, and microbial metabolites.^12^ Large cohort studies and limited interventional studies have correlated fermented food consumption with weight maintenance and reduced risk of diabetes, cancer and cardiovascular disease.^13,14^ A recent study found differences in gut microbial structure and metabolome between consumers of fermented and non-fermented foods.^15^ The benefits of a fermented diet on gut and host health have been reported,^16^ while the effects of a maternal fermented diet (MFD) on gut health in offspring remain undetermined.

In the present study, *in vivo* and *in vitro* models were used to investigate how MFD protects against early-life intestinal inflammation by applying multi-omics, fecal microbiota transplantation (FMT), and lipopolysaccharide (LPS)-induced colonic inflammation. These results will expand our knowledge of how MFD influences the gut health of infants during early life and provide underlying strategies to prevent early-life gut inflammatory injury with lasting consequences.

## Materials and methods

### Production of maternal fermented diet

The substrate for fermentation consisted of corn, soybean meal, and wine lees (2:2:1). Sterile water was added to achieve an optimal moisture content of 40%. *Bacillus subtilis* CW4 (NCBI Accession No. MH885533, 1 × 10^8^ CFU/g) and *Enterococcus faecalis* CWEF (NCBI Accession No. MN038173, 1 × 10^8^ CFU/g) were included in the diet to promote fermentation over the course of 3 days. Moist samples (approximately 100 g) were collected to determine the numbers of microorganisms and 16S rRNA gene sequencing, and the remainder of the sample material was dried at 60°C for 24 h, cooled, ground, and subjected to conventional nutrient analysis. Dried samples were collected for further analysis of the crude protein (CP), neutral detergent fiber (NDF), acid detergent fiber (ADF) and amylose content using the AOAC International guidelines.^17^ Lactate was detected using a lactic acid assay kit (Nanjing Jiancheng Bioengineering Institute, Nanjing) following the manufacturer’s instructions. The nutrient content of the MFD is shown in Table S1.

### Experimental design

#### In vivo

All the animal assays were approved by the Institutional Animal Care and Use Committee at Zhejiang University.

**Figure 1.**
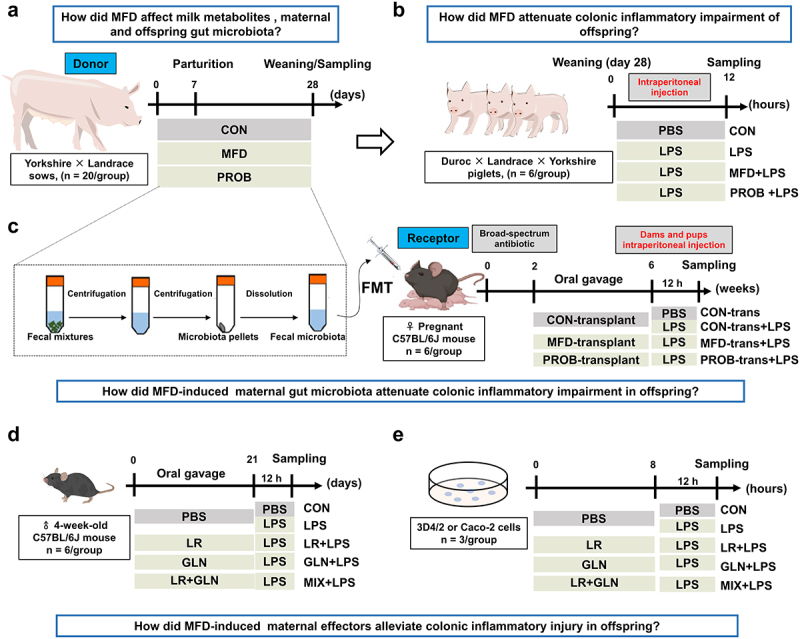
Study design for the whole experiment. (a) Schematic diagram of the fermented diet and probiotics administered to sows (n = 20). (b) Schematic diagram of the susceptibility of piglets to LPS-induced colonic inflammation (n = 6). (c) Schematic diagram of the effects of the transplantation of sows’ fecal microbiota on gut health in mouse dams and pups (n = 6). (d) Schematic diagram of the effects of LR and GLN on the gut health of mice (n = 6). (e) Schematic diagram of the effects of LR and GLN on inflammation in 3D4/2 and Caco-2 cells (n = 3). PBS, phosphate-buffered saline; LPS, lipopolysaccharides; FMT, fecal microbiota transplantation; LR, *Lactobacillus reuteri*; GLN, L-glutamine.

Sixty Yorkshire × Landrace sows were randomly divided into three groups and fed different diets from 7 days before parturition to the day of weaning (Figure 1a). The groups were as follows: (i) control (CON) group (control diet, n = 20), (ii) MFD group (basal diet + 10% MFD, n = 20) and (iii) probiotic (PROB) group (basal diet + equal amounts of *B. subtilis* and *E. faecalis*, n = 20). For the PROB group, 3.4 g/kg of *B. subtilis* powder and 1.3 g/kg of *E. faecalis* powder were supplied in the drinking water. The diets had the same amounts of crude protein and digestive energy and met the NRC (2012) nutrient requirement.^18^ The ingredients and nutritional values are shown in Table S3. Sow feces were collected on day 28, and piglet feces were obtained on days 7, 14 and 28 (with the count beginning 7 days before parturition).

To examine how MFD affects colonic inflammation in offspring, piglets were challenged with lipopolysaccharide (LPS, Sigma–Aldrich, St. Louis, MO, USA) (Figure 1b). On day 28, healthy piglets with similar weights were randomly selected from the CON, MFD, and PROB groups. Piglets in each group were intraperitoneally injected with 10 mg/kg LPS (n = 6) ^19^ for 12 h or, in the case of six piglets in the CON group, an equivalent amount of PBS (n = 6). Piglets were divided into CON, LPS, MFD + LPS, and PROB + LPS groups; slaughtered and sampled 12 h after LPS challenge.

To further investigate whether an MFD could affect early-life gut health by regulating the maternal gut microbiome, FMT was performed in a mouse model (Figure 1c). Twenty-four healthy, pregnant, specific-pathogen-free (SPF) female C57BL/6J mice were divided into four groups, with 6 mice per group. After 2 weeks of broad-spectrum antibiotic treatment (50 µg/mL clindamycin, 50 µg/mL metronidazole, 50 µg/mL penicillin, 25 µg/mL vancomycin and 50 µg/mL neomycin (Sigma, St Louis, USA)) in sterile drinking water, the dams were intragastrically dosed with a sow fecal microbiota suspension every other day (n = 6). At the time of weaning on day 21, the dams and pups (n = 6) were intraperitoneally injected with 10 mg/kg LPS, and feces and colon samples were collected 12 h later. Dams and pups were divided into CON-trans, CON-trans + LPS, MFD-trans + LPS and PROB-trans + LPS groups.

Mice were further used to verify the properties of maternal effectors *in vivo* (Figure 1d). Thirty healthy 4-week-old SPF C57BL/6J male mice were allotted into five groups (n = 6). They were pre-fed for one week and treated with 1 ×10^8^ CFU/d *Lactobacillus reuteri* (LR, ATCC 53608), 300 mg/kg L-glutamine (GLN, Solarbio, Beijing, China), or a combination of the two agents (MIX) for 21 days. Thereafter, the mice were intraperitoneally injected with 10 mg/kg LPS for 12 h, and fecal and colon samples were collected. The groups were CON, LPS, LR + LPS, GLN + LPS, and MIX + LPS.

#### In vitro

Porcine 3D4/2 macrophages and human Caco-2 cells were selected to investigate the effects of maternal effectors on LPS-induced inflammation *in vitro* (Figure 1e). Cells were treated with 10 µg/mL LPS or an equivalent amount of PBS for 12 h after treatment (optimal concentration: 1 ×10^8^ CFU/mL LR or 2.5 mM GLN) for 8 h (n = 3). Then, the total protein of the cells were collected by using a total protein extraction kit (KeyGen BioTECH, Nanjing, China).

### 16S sequencing and data analysis

Microbial DNA was obtained from fecal homogenates using the E.Z.N.A. Stool DNA Kit (Omega Bio-Tek, Norcross, GA, USA). To ensure that no contamination had occurred, the concentration and purity of the DNA samples were measured with a NanoDrop 2000 UV–vis spectrophotometer (Thermo Scientific, Wilmington, MA, USA) and examined by electrophoresis using 1% agarose gels. The V4 gene regions of the bacterial 16S rRNA gene were amplified with the primer pairs 515 F (5’-GTGCCAGCMGCCGCGG-3’) and 806 R (5’-GGACTACHVGGGTWTCTAAT-3’) using the Illumina MiSeq platform at Shanghai Majorbio Biopharm Technology Co., Ltd. (Shanghai, China).

The reads were preprocessed, denoised, quality controlled, and merged in DADA2.^20^ UPARSE 7.1 and QIIME2ʹs q2-feature-classifier plugin were used to cluster the operational taxonomic units (OTUs) with 97% and 100% similarity cutoffs,^21^ respectively, and chimeric sequences were identified and removed. The taxonomies from the phylum to genus levels were assigned by the Greengenes database.^22^ Sequences of OTUs of interest were further confirmed through NCBI BLAST analysis. Diversity analyses were performed using QIIME1 and QIIME2.^23^ In addition, the significantly different genera were selected by the linear discriminant analysis effect size (LEfSe) method (https://huttenhower.sph.harvard.edu/galaxy/).^24^ To predict metabolic genes during the process, PICRUSt 1 and 2 were used to obtain functions for the genes that were predicted to be present in the samples and to assign the genes to Kyoto Encyclopedia of Genes and Genomes (KEGG) metabolic pathways.^25^ Pearson’s rank correlation coefficient was calculated with R version 3.6.3 to evaluate the relationships among the data. To identify age-related bacteria in the gut microbiota, a random forest model was used to train the data in the control group, and then the age of the microbial community was modeled for those same taxa in all groups. The maturation index of the intestinal microbiota was calculated by the method described in a previous article.^26^ The significangtly different microbes of the MFD are shown in Table S3.

### Metabolomic profiling by LC-TOF/MS

Milk samples randomly selected from six sows in each group were used for extraction and sent for metabolomic analysis (Shanghai Biotree Biotech Co., Ltd., Shanghai, China). An equivalent volume was aliquoted from each sample, mixed to prepare the quality control sample and dried in a vacuum concentrator. In addition, methoxymethyl amine salt was mixed with the dried samples, and bis(trimethylsilyl)trifluoroacetamide was added. After the samples cooled to room temperature, fatty acid methyl ester was added to each sample and mixed. Ultra-high-performance liquid chromatography (1290 Infinity series UHPLC System, Agilent Technologies, Santa Clara, CA, USA) was applied for LC-TOF/MS analysis. A UPLC BEH amide column (internal diameter, 2.1 × 100 mm, 1.7 μm, Waters, Milford, MA, USA) was used for separation. The obtained data were applied for sparse partial least squares discriminant analysis (sPLS-DA). Differential metabolites were identified with variable importance projection (VIP) > 1.0 and *P* < 0.05. To further interpret the biological significance of metabolites, metabolic pathway analyses were performed by an online analysis platform in MetaboAnalyst 5.0 (https://www.metaboanalyst.ca/). KEGG analysis was conducted using the enrichment analysis sections of MetaboAnalyst.

### Intestinal morphology and histology

Approximately 2 cm of the proximal colon was fixed in 4% paraformaldehyde. Colonic tissues were stained with hematoxylin and eosin. The Leica DM3000 Microsystem, and Leica Application Suite 3.7.0 (Leica, Wetzlar, Germany) were applied to observe the colonic morphology. Histological scoring was performed as described by Geng et al.^19^

A colonic tissue sample of suitable size was placed in 2.5% glutaraldehyde and fixed overnight. The fixed sample was washed with PBS, soaked in 1% osmic acid solution for 2 h and washed with PBS. Subsequently, the sample was dehydrated in different concentrations of alcohol and dehydrated twice with absolute ethanol. The sample was transferred to a 1:1 volume mixture of ethanol and isoamyl acetate for 30 min and then transferred to pure isoamyl acetate for 1 h. After the critical point drying, the samples were coated with gold-platinum film and observed with a field emission scanning electron microscope.

The colonic tissue fixed in the osmic acid solution was washed with PBS and dehydrated in different concentrations of alcohol and acetone. The sample was placed in a mixture of pure acetone and embedding agent for 1 h, transferred to a mixture of pure acetone and embedding agent for 3 h, and finally transferred to embedding agent for 12 h. The embedded sample was heated and then sectioned with an ultrathin microtome. Finally, the sections were stained and treated with uranyl acetate and alkaline lead citrate. A suitable field of view was identified and imaged with a JEM-1011 transmission electron microscope (JEOL USA, Peabody, MA, USA).

### Immunofluorescent staining and TUNEL

Colonic slides were incubated with antigen recovery solution (Vector Laboratories, Inc. Burlingame, CA, USA) for 15 min, and nonspecific binding was blocked with 5% BSA. Next, the slides were incubated with anti-ZO-1 and anti-β-catenin primary antibodies (ab96587 and ab32572, Abcam, Cambridge, MA, USA) at 4°C overnight. After being washed with PBS, slides were incubated with fluorescent dye-conjugated secondary antibodies (Abcam) for 1 h and protected from light. Finally, 4,6-diamidino-2-phenylindole (DAPI) was used to stain the nucleus. Sections were examined with a DM5000 B fluorescence microscope (Leica, Wetzlar, Germany).

After the colonic sections were deparaffinized, DNase-free proteinase K (20 μg/mL) was applied, and the sections were incubated at 37°C for 20 min. After washing with PBS, the TUNEL detection solution was added dropwise, and sections were incubated in a 37°C incubator for 2 h. After the stop solution was added to terminate the reaction, the sections were washed with PBS. Thereafter, the streptavidin-HRP working solution was added dropwise, and sections were incubated for 30 min. The sections were washed with PBS, then treated with 0.2–0.5 mL of DAB and incubated at room temperature. The specific time was determined according to the degree of color development, and images were acquired for analysis.

### Western blotting

Total protein was obtained using a Protein Extraction Kit (KeyGen BioTECH, Nanjing, China). SDS–PAGE separates proteins of different sizes and electroporates them onto PVDF membranes (Millipore, Bedford, MA, USA). Membranes were blocked with 5% skimmed milk and incubated with anti-ZO-1(ER41204), anti-Occludin(R1510-33), anti-Claudin 1(ER1906-37), anti-Cleaved caspase 3(ET1602-47), anti-Bax(ER0907), anti-Bcl-2(ER1802-97), anti-iNOS(ER1706-89), anti-Arg1(ET1605-8), anti-p-p38(ER2001-52), anti-p38(ET1602-26), anti-p-JNK(ET1609-42), anti-JNK(ET1601-28), anti-p-ERK1/2(ET1603-22), anti-ERK1/2(ET1601-29), and anti-β-actin(R1207-1) antibodies at 4°C overnight(HUABIO, Hangzhou, China). After being washed with Tris Buffered Saline with Tween20 (TBST), the membranes were incubated with secondary antibodies for 1 h. Protein bands were visualized with an enhanced chemiluminescence (ECL) assay kit (Biosharp, Hangzhou, China) and measured with ImageJ software (NIH, Bethesda, MD, USA).

### ELISA

Approximately 0.1 g of colonic tissue was homogenized after being centrifuged at 12,000 × *g* for 10 min at 4°C. Cytokine (IL-β, IFN-γ, IL-6, IL-10, TNF-α, TGF-β) concentrations in supernatants were measured with ELISA kits (Jiangsu Meibiao Biological Technology Co., Ltd., Jiangsu, China).

#### *In vitro* fermentation

Approximately 1 g of predigested MFD was suspended in 25 mL of sterile fermentation medium and boiled for 10 min. The boiled MFD solution was introduced into the anaerobic chamber, cooled to room temperature, and reduced for 2 h. Thereafter, 5 mL of sterile fermentation medium was combined with 0.1 g of fecal sample and homogenized, followed by the mixing of 2.5 mL of the homogenized fecal suspension and 2.5 mL of the MFD solution and incubation under anaerobic conditions at 37°C with 125 rpm shaking for 14 h. All fermentation steps were conducted in an anaerobic chamber (Bactron, Sheldon Manufacturing, Inc., Cornelius, OR, USA) in an anaerobic environment containing 5% CO_2_, 5% H_2_, and 90% N_2_.

### Quantitative real-time PCR and absolute quantification of *L.* *reuteri*

Primers were used to measure the copy numbers of the 16S rRNA gene of specific bacteria (V3-V4). The *Lreu* gene was selected to analyze the abundance of *L. reuteri*. Forward and reverse primers were used as follows: 5′-CAGACAATCTTTGATTGTTTAG-3′ and 5′-GCTTGTTGGTTTGGGCTCTTC-3′.^27^ To obtain a standard curve, plasmids containing target DNA PCR fragments of 16S rRNA and *Lreu* genes were diluted 10-fold. qPCR and analysis were conducted as previously described.^24^ The ^Δ^Ct method was used to calculate the copy numbers of *Lreu* genes from the standard curve of 16S rRNA.

### Cell culture

Caco-2 and 3D4/2 cells were obtained from ATCC Cell Bank (Shanghai, China). DMEM-F12 and RPMI-1640 medium supplemented with 10% fetal bovine serum and two antibiotics (100 U/mL penicillin and 100 µg/mL streptomycin sulfate) in 6- and 12-well plates at 37°C was used for Caco-2 and 3D4/2 cells, respectively. Cells were incubated in an incubator (Thermo Fisher Scientific, MA, USA) containing 5% CO_2_ until they reached approximately 80% confluence before the treatments. The specific activators of p38 (S2266), JNK (S7409), and ERK1/2 (S1013) were purchased from Selleck (TX, USA).

### Transepithelial electrical resistance (TER) and *in* *vitro* gut paracellular permeability

Transwell filters (Corning, NY, USA) were used to culture Caco-2 cells. When the TER stabilized, the cells were used for subsequent experiments. The TER was determined using a Millicell ERS Voltohmmeter (Millipore, Burlington, MA, USA) at 0, 2, 4, 6, 8, 10, and 12 h after LPS stimulation. Changes were analyzed as a fold of TER at 0 h. Approximately 100 µL of 4-kDa FD4 (1 mg/mL) was added to the apical chamber after the last TER measurement. The plates were placed in a humidified incubator for 30 min. The prepared FD4 solution was serially diluted to produce concentrations of 5, 10, 20, 40, 80, 160, 320, 640, and 1,280 ng/mL. Subsequently, the level of FD4 in the basolateral chamber was measured (excitation wavelength, 492 nm; emission wavelength, 520 nm).

### Statistical analysis

The nutritional value of the MFD, animal performance and molecular biological data are presented as the mean ± standard error of the mean as analyzed by SPSS 26.0 (SPSS Inc. Chicago, IL, USA) using one-way ANOVA and Duncan’s multiple tests to compare the significant differences. The “corrplot” package in R (R Core Team, 2014) was used to obtain the Pearson correlation coefficient and significance. Differential gut microbes and milk metabolites were verified by ANOVA and Benjamini–Hochberg FDR adjustment in STAMP (version 2.1.3). The contribution of maternal factors to the gut microbiota in offspring was calculated by variance partitioning analysis (VPA) using the “varpart” package of R.

## Results

### MFD improved the performance of sows and piglets

Swine (*Sus scrofa*), as an animal biomedical model, have been extensively used to study the gut microbiota, its host interactions, and the consequences for gut health because their gut is physiologically and structurally similar to that of humans and because their diets are easily manipulated. The nutritional value and microbe content of the experimental MFD are presented in Tables S1 and S3. The levels of crude protein, small peptides, lactic acid and probiotics significantly increased, after fermentation, while the levels of NDF, ADF and amylose markedly decreased (*P* < 0.05). *Enterococcus, Bacillus* and *Pseudomonas* were the three most enriched genera in the MFD. The impact of the MFD on the performance of sows and piglets was specifically examined. As shown in Table S4, the MFD significantly improved the average daily feed intake (*P* = 0.01) and milk yield (*P* = 0.03) in sows. Piglets in the MFD group showed a significant increase in weaning weight gain (*P* = 0.04) and a decrease in diarrhea incidence (*P* = 0.00). Probiotic treatment tended to increase the average daily feed intake and milk yield in sows, as well as piglet weaning weight gain, and notably decreased the incidence of diarrhea (*P* = 0.03).

### MFD changed the composition of the sow gut microbiota

**Figure 2.**
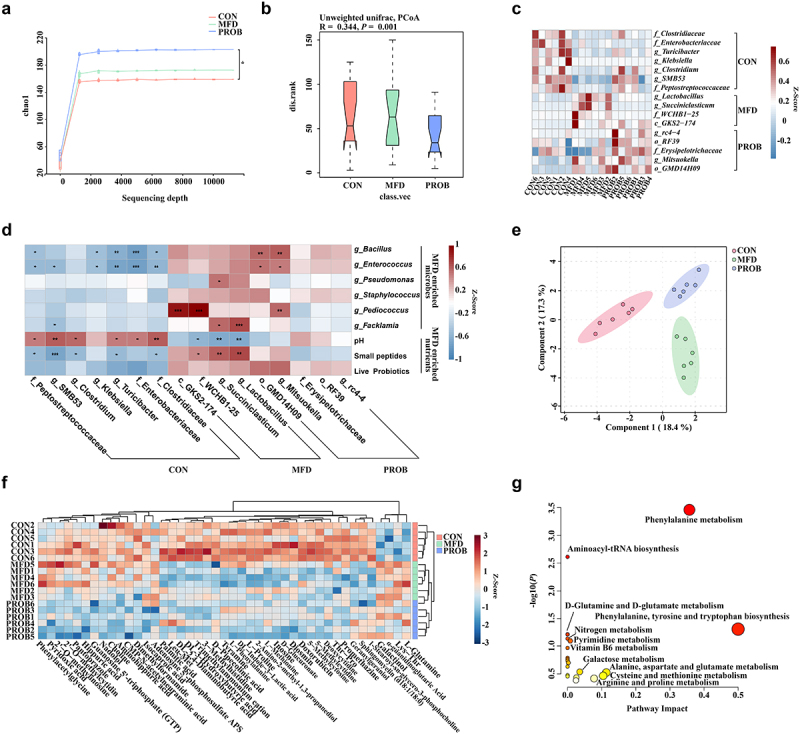
The effects of MFD on the gut microbiota and milk metabolome of sows (n = 6). (a) Chao1 index dilution curve of α-diversity. (b) Principal coordinates analysis (PCoA) boxplot of β-diversity based on the Unweighted unifrac distance (ANOSIM analysis, *P* < 0.001). (c) Distribution of significantly different gut microbe genera in individual sows (LEfSe analysis, LDA > 3.0). (d) Correlations between the MFD and sow gut microbiota. (e) sPLS-DA plot of maternal milk metabolites (ANOSIM analysis, *P* < 0.001). (f) Distribution of differential milk metabolites in individual sows based on Pearson distance and average clustering (VIP > 1.0, *P* < 0.05). (g) The significantly different metabolite-related metabolic pathways of milk metabolites.   ***: *P* < 0.001, **: 0.001 < *P* < 0.01, *: 0.01 < *P* < 0.05 indicate significance.

Regarding the maternal aspects, the effects of the MFD on the gut microbiota in sows were investigated. Compared with the CON group, the α-diversity of the maternal gut microbiota in the MFD group on day 28 had a trend-level increase, whereas it was significantly increased in the PROB group (*P* = 0.04) (Figure 2a). Principal coordinates analysis (PCoA) analysis showed that the β-diversity of the maternal gut microbiota on day 28 was significantly different among the groups (Figure 2b, *P* = 0.001). A heatmap of the most differentially abundant genera revealed that the abundance of gut *Lactobacillus* and *Succiniclasticum* significantly increased in the MFD group on day 28, whereas the abundance of *Mitsuokella* and *Erysipelotrichaceae* increased in the PROB group (Figure 2c, LDA > 3.0). The MFD enhanced the functions of sow gut microbiota related to the metabolism of carbohydrates, proteins, and fats (Fig. S1a). To explain the impacts of MFD on the gut microbiota of sows, we analyzed the correlations of the significantly different nutritional and microbial content of the MFD with the sow gut microbiota (Figure 2d). The results indicated that small peptides and the pH of the MFD significantly induced the abundance of sow gut *Lactobacillus and Succiniclasticum* (*P* < 0.05).

### MFD changed the composition of the sow milk metabolome

MFD also changed the milk metabolome in sows. The sPLS-DA plot of cluster analysis showed that the clusters between the three groups were clearly separated (Figure 2e, *P* < 0.001). Among the 43 differential metabolites, MFD significantly increased the concentrations of α-guanidine glutaric acid, hippuric acid, L-glutamine (GLN), and pyridoxic acid in the milk but significantly reduced the concentrations of geranylgeraniol, 1-Stearoyl-sn-glycero-3-phosphocholine, deoxycytidine, trimethylammonium cation, and 5-methylcytosine (Figure 2f, variable importance projection (VIP) > 1.0, *P* < 0.05). The significantly different metabolites were taken to indicate the significantly different metabolic pathways reflected in the milk metabolome (Figure 2g, *P* < 0.05). Phenylalanine metabolism; phenylalanine, tyrosine and tryptophan biosynthesis; aminoacyl-RNA biosynthesis; D-glutamine and D-glutamate metabolism; and alanine, aspartic acid and glutamate metabolism were significantly different among the groups. Among them, D-glutamine and D-glutamate metabolism, aminoacyl-RNA biosynthesis, and phenylpropane metabolism were the top three significantly enriched metabolic pathways. The top 15 significantly different metabolites and metabolite-related metabolic pathways of sow’s milk are shown in Fig. S1b and c.

### MFD improved the longitudinal and horizontal assembly of early-life gut microbiota in piglets

**Figure 3.**
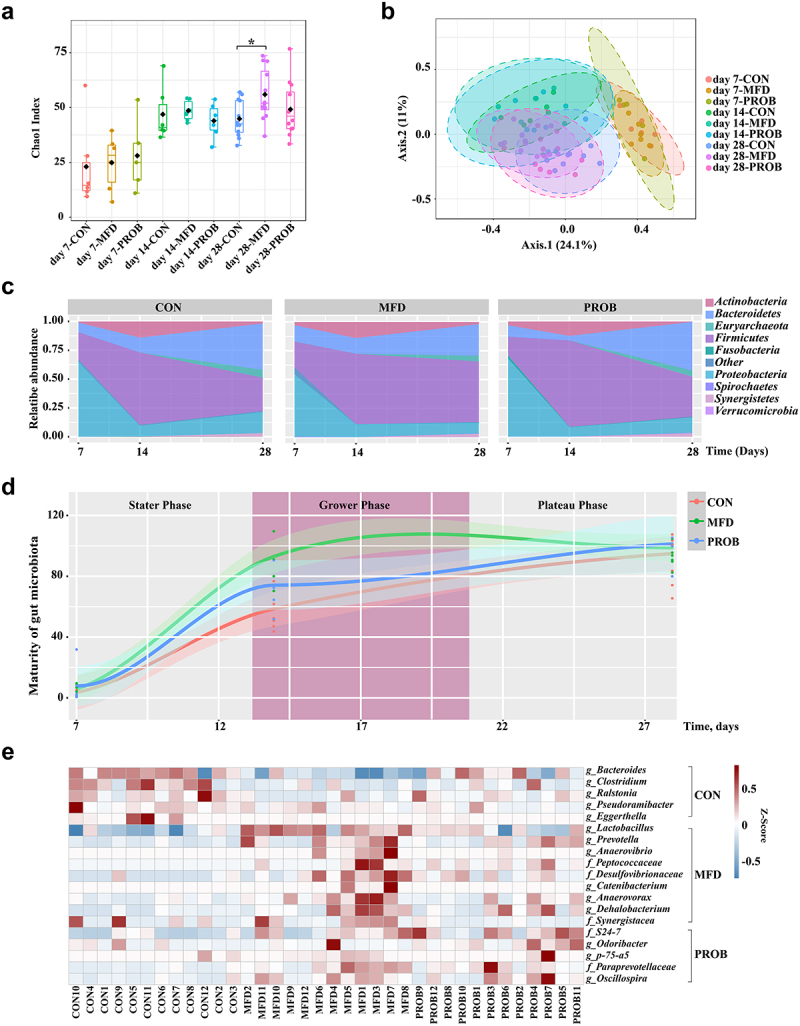
The effects of MFD on the longitudinal and horizontal assembly of early-life gut microbiota in piglets (n = 6). (a) Chao1 index of α-diversity. (b) PCoA plots of β-diversity based on the Bray–Curtis index (ANOSIM analysis, *P* < 0.001). (c) Area plots at the phylum on days 7, 14 and 28. (d) Maturation curve of gut microbiota. (e) Distribution of significantly different gut genera in offspring individuals on day 28 (LEfSe analysis, LDA>3.0).

The early gut microbiota is essential for gut development and health. Therefore, the effects of the MFD on the longitudinal and horizontal assembly of the gut microbiota in piglets were investigated. The α-diversity of the gut microbiota of piglets continued to increase as aging during lactation (Figure 3a). There were no differences of the α and β-diversity of the gut microbiota on day 7 and 14, but there were significant differences on day 28 (Figure 3b, Fig. S2b, *P* < 0.05). At the phylum level, the MFD increased and then maintained the abundance of gut *Firmicutes* in piglets, whereas this taxon showed an increasing trend followed by a decreasing trend in other groups (Figure 3c). Interestingly, the maturation patterns of the gut microbiota, as indicated by the microbiota maturation index of each group, were different (Figure 3d). The piglet gut microbial development curve of the MFD was the fastest group, reaching a plateau as early as day 14 of lactation. To better understand how the MFD significantly affects interactions between microbes in the gut microbiota of piglets, the co-occurrence network of gut microbial interactions at different time points was analyzed. The abundance and correlations with other taxa (edge number) of *Lactobacillus, Bifidobacterium, Prevotella* and *Oscillatoria* were higher than those of other genera (Fig. S3). In addition, MFD significantly reduced the significance of LPS biosynthesis and bacterial toxins of piglet gut microbiota over time (Fig. S2b). The gut microbiota and functions of the three groups of piglets on day 28 were further analyzed. Compared to the other groups, the MFD increased the abundance of gut *Lactobacillus* in piglets (Figure 3e, LDA > 2.5) and metabolic processes such as those involving amino acid-related enzymes and glucose metabolism (Fig. S2c).

### MFD reduced the susceptibility of piglets to LPS-induced colonic inflammation

**Figure 4.**
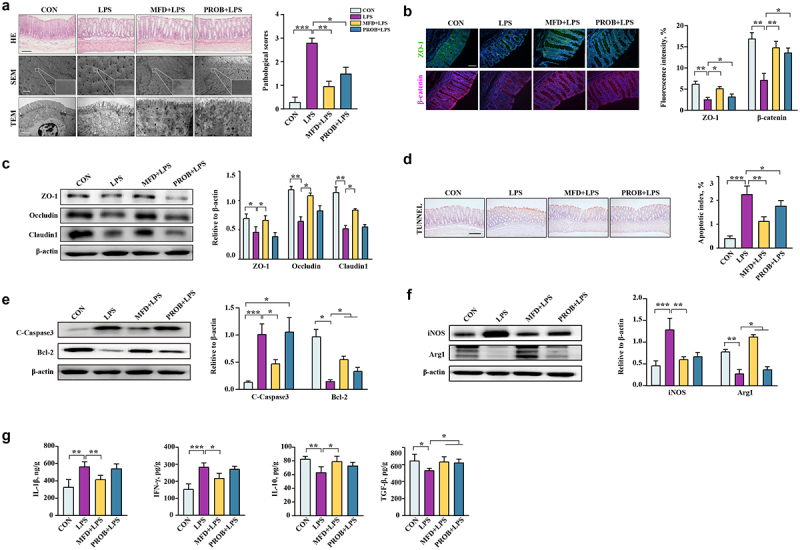
The impacts of an MFD on the susceptibility of piglets to LPS-induced colonic epithelial inflammation (n = 6). (a) Colonic histological morphology images and pathological scores. Scale bar of hematoxylin and eosin staining images: 200 μm; scale bar of scanning electron microscope (SEM) images: 1 μm; scale bar of transmission electron microscope (TEM) images: 0.2 μm. (b) Images of immunofluorescence of ZO-1 and β-catenin. (c) Colonic tight junction protein expression. (d) TUNEL image and statistical analysis. (e) Apoptosis-related protein expression in the colon. (f) M1 and M2 macrophage surface protein expression in colon. (g) Colonic cytokines. ***: *P* < 0.001, **: 0.001 < *P* < 0.01, *: 0.01 < *P* < 0.05 indicate significance.

Neonates are sensitive to pathogenic infection because their digestive system is not yet completely developed, making them prone to colonic inflammation. To assess the resistance of offspring to colonic inflammation, piglets were challenged with LPS. The results of hematoxylin and eosin staining and electron microscopy revealed that the MFD significantly alleviated the LPS-induced injury of the piglet colon, that is, the morphology of the microstructure (Figure 4a, *P* < 0.05). Similarly, the MFD restored the decreased protein levels of ZO-1, β-catenin, Occludin, and Claudin 1 in the piglet colon induced by LPS (Figure 4b, c, *P* < 0.05). In addition, a decrease in the colonic apoptotic index and cleaved caspase 3 protein expression , and an increase in Bcl2 protein expression in piglets of the MFD group were observed compared to the LPS group (Figure 4d, e, *P* < 0.05). In terms of colonic macrophage status, the results showed that the MFD alleviated the decrease in Arg1 and increase in iNOS caused by LPS (Figure 4f, *P* < 0.05). The MFD alleviated the LPS-induced increase in IL-1β and IFN-γ levels and the decrease in IL-10 and TGF-β levels in the colon (Figure 4g, *P* < 0.05). The overall mitigatory effects were greater in the MFD group than in the PROB group.

### MFD programmed the early-life gut health of offspring by altering the maternal gut microbiota

**Figure 5.**
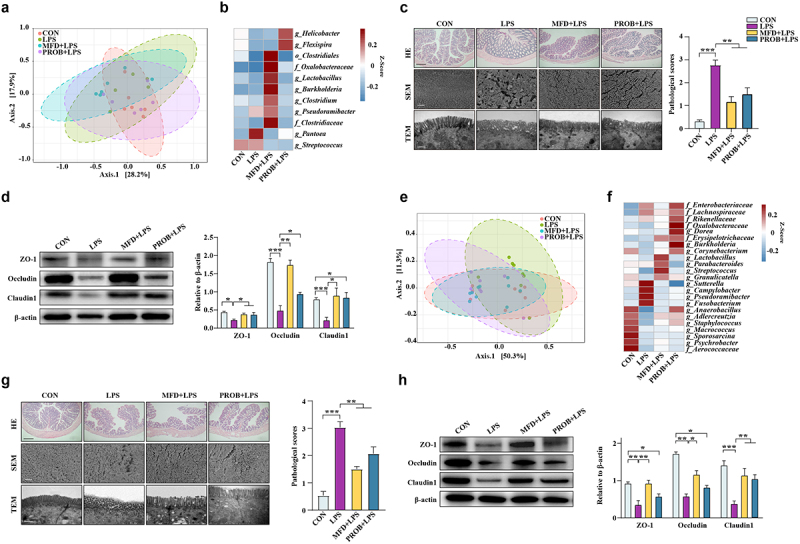
The effects of maternal FMT from MFD-fed sows on the susceptibility of mouse dams and pups to LPS-induced colonic epithelial inflammatory impairment (n = 6). (a) β-diversity of dam gut microbiota based on Bray–Curtis index (ANOSIM analysis, *P* = 0.049). (b) Distribution of differentially abundant genera in the gut of mouse dams (LEfSe analysis, LDA>3.0). (c) Colonic histological morphology images of dams and pathological scores. Scale bar of hematoxylin and eosin staining images: 200 μm; scale bar of scanning electron microscope (SEM) images: 1 μm; scale bar of transmission electron microscope (TEM) images: 0.2 μm. (d) Colonic tight junction protein expression of dams. (e) β-diversity of pup gut microbiota based on Bray–Curtis index (ANOSIM analysis, *P* = 0.007). (f) Distribution of differentially abundant genera in the gut of mouse pups (LEfSe analysis, LDA>2.5). (g) Colonic histological morphology images of pups and pathological scores. Scale bar of hematoxylin and eosin staining images: 200 μm; scale bar of scanning electron microscope (SEM) images: 1 μm; scale bar of transmission electron microscope (TEM) images: 0.2 μm. (h) Colonic tight junction protein expression of pups. ***: *P* < 0.001, **: 0.001 < *P* < 0.01, *: 0.01 < *P* < 0.05 indicate significance.

To verify whether the MFD improves early-life gut health by regulating the maternal gut microbiota, FMT assay was performed. Fecal microbiota samples from sows of the CON, MFD, and PROB groups were transplanted to antibiotic-treated mouse dams for 4 weeks. The mouse dams and pups were intraperitoneally injected with LPS on the weaning day of pups. The β-diversity of the gut microbiota of mouse dams transplanted with sow fecal microbiota was significantly different after LPS treatment (Figure 5a, *P* = 0.049). The abundance of gut *Lactobacillus* and *Clostridium* in mouse dams in the MFD-trans group was remarkably enriched compared to that in the other groups, whereas the abundance of *Streptococcus, Pantoea*, and *Flexispixa* was enriched in the CON, LPS, and PROB groups, respectively (Figure 5b, LDA > 3.0). With regard to the morphology of the gut in mouse dams, maternal FMT from the MFD group alleviated LPS-induced colonic injury and decreased ZO-1, Occludin, and Claudin 1 protein levels (Figure 5c, d, *P* < 0.05). The results of mouse pups after LPS treatment were similar to those of dams. However, there was no difference in the weaning weight among the groups (Fig. S4a). An increasing tendency was found in the weaning weight gain of pups (Fig. S4a, *P* = 0.067). Maternal FMT markedly differentiated the β-diversity of the gut microbiota in mouse pups after LPS challenge (Figure 5e, *P* = 0.007). The abundance of gut *Lactobacillus* and *Streptococcus* in mouse pups of the MFD-trans group was notably enriched compared with that in the other groups after LPS treatment, whereas the abundance of *Anaerobacillus, Sutterella*, and *Burkholderia* was enriched in the CON-trans, CON-trans+LPS, and PROB-trans+LPS groups, respectively (Figure 5f, LAD > 2.5). The LPS-induced injury to the morphology of the colon, the increase in gut permeability, and the decline in ZO-1, Occludin and Claudin 1 protein levels in pups were reversed in the MFD transplanted group (Figure 5g, h; Fig. S4b, *P* < 0.05). The transplantation of fecal microbiota from the MFD mitigated the LPS-induced increase in serum DAO, DLA, colonic IL-6 and TNF-α and the decrease in colonic IL-10 of pups (Fig. S4c, *P* < 0.05). The overall colonic status of both mouse dams and pups in the MFD-trans group after LPS challenge was better than that of the PROB-trans group.

### Sow’s gut Lactobacillus reuteri and milk L-GLN are two MFD-induced effectors that enhance gut health in piglets

**Figure 6.**
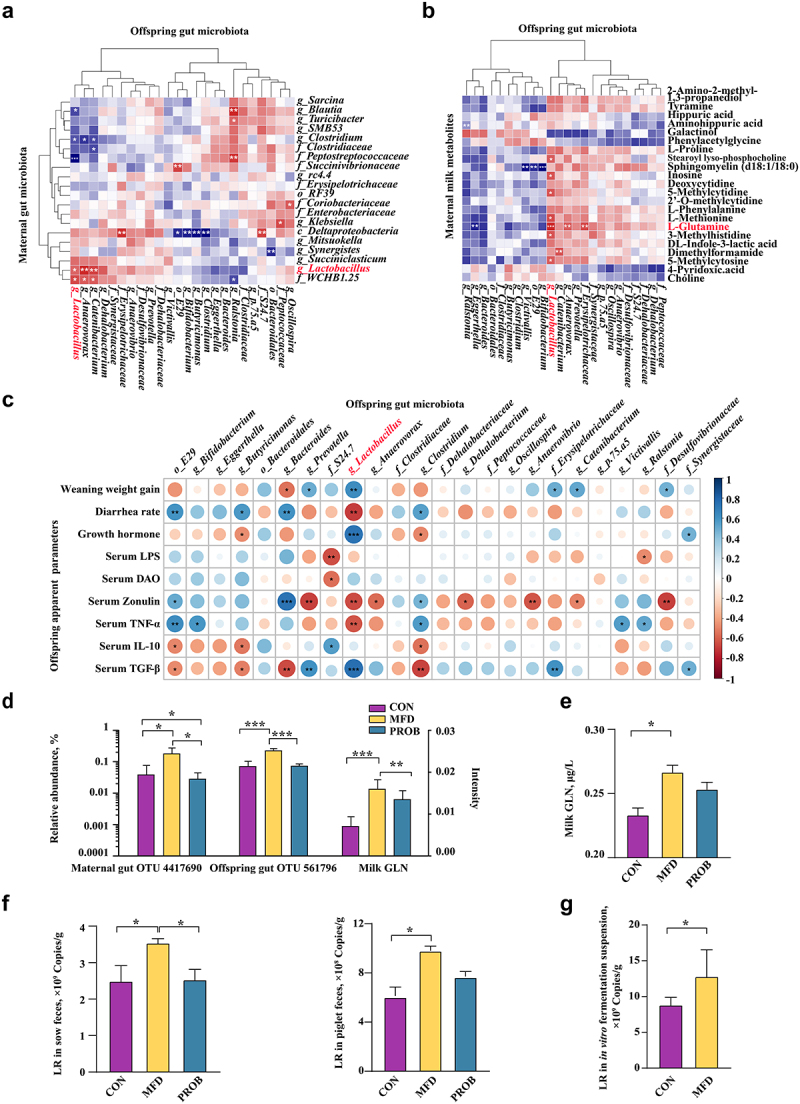
The sow’s effectors that contribute to piglets gut health (n = 6). (a) Correlations between sow gut microbiota and piglet gut microbiota based on Pearson coefficient. (b) Correlations between milk metabolites and piglet gut microbiota based on Pearson coefficient. (c) Correlations between piglet gut microbiota and piglet serum indexes based on Pearson coefficient. (d) The relative abundance of the most differentially abundant OTUs in both the sow and piglet gut microbiota and the concentration of GLN in the milk. (e) The absolute concentration of milk GLN. (f) The absolute quantity of fecal LR of sow and piglets. (g) The absolute quantity of LR in thesupernatant of the MFD in vitro fermentation system. ***: *P* < 0.001, **: 0.001 < *P* < 0.01, *: 0.01 < *P* < 0.05 indicate significance.

Maternal gut microbiota and milk play critical roles in early-life gut health. The neonatal gut microbiota is essential in the maintenance of gut health. Therefore, correlation analysis was performed to investigate the effectors of sow’s gut microbiota and milk metabolites that affected the gut health of piglets. The results indicated that there was a significant positive correlation between gut *Lactobacillus* levels in sows and piglets (Figure 6a, *P* < 0.05). A significant positive correlation between GLN in the milk metabolite fraction and gut *Lactobacillus* in piglets was also identified (Figure 6b, *P* < 0.05). Next, the main maternal variables that shaped the gut microbiota of piglets were explored. The variation of piglets’ gut microbiota explained by the sow gut microbiota and milk metabolites were 0.86% and 1.36%, respectively (Fig. S5). The combined explanation of both sow gut microbiota and milk metabolites reached 93.52%, and the unexplainable factor accounted for 4.26%. The result suggested that the combined effects of sow gut microbiota and milk metabolites were the most important variables for gut microbiota assembly in piglets. Additionally, correlation heatmap analysis of piglet gut microbiota and piglet phenotypic indices showed that gut *Lactobacillus* was significantly positively correlated with piglet weaning weight gain, serum growth hormone, and TGF-β concentration and negatively correlated with the diarrhea rate, zonulin expression, and TNF-α concentration (Figure 6c, *P* < 0.05). In addition, the relative abundance of gut *Lactobacillus* in sows and piglets and the concentration of GLN in the milk metabolite fraction were enriched in the MFD group (Figure 6d, *P* < 0.05). The absolute concentration of milk GLN increased in the MFD group (Figure 6e, *P* < 0.05). The sequences of the most significantly different OTUs of gut *Lactobacillus* in sows and piglets were BLASTed against the NCBI database and found that *Lactobacillus reuteri* (LR) has a high score suggesting this is the most likely taxa associated with the OTU (Table S5, 6). In addition, the absolute quantification of LR in sow and piglet feces was enriched in the MFD group (Figure 6f, *P* < 0.05). Furthermore, the *in vitro* fermentation of maternal fecal microbiota from the CON group indicated that the MFD could enhance the proliferation of LR compared to the CON diet (Figure 6g, *P* < 0.05).

### LR and GLN alleviated LPS-induced colonic inflammation by suppressing phosphorylation in the p38 MAPK/JNK pathway

**Figure 7.**
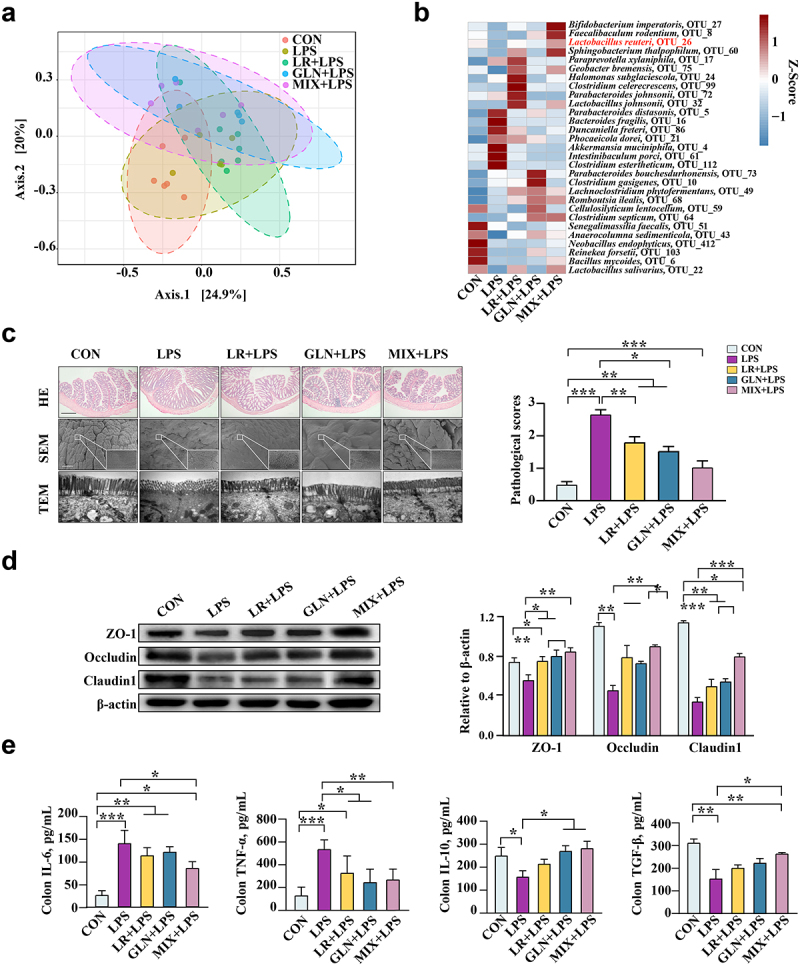
Validation of the effects of LR and GLN on LPS-induced inflammatory injury in the colon of mice (n = 6). (a) β-diversity of gut microbiota based on Bray–Curtis index (ANOSIM analysis, *P* < 0.001). (b) Distribution of differentially abundant gut microbe genera in mice (LEfSe analysis, LDA>3.0). (c) Colonic histological morphology images and pathological scores. Scale bar of hematoxylin and eosin staining images: 200 μm; scale bar of scanning electron microscope (SEM) images: 1 μm; scale bar of transmission electron microscope (TEM) images: 0.2 μm. (d) Colonic tight junction protein expression. (e) Colonic cytokines. ***: *P* < 0.001, **: 0.001 < *P* < 0.01, *: 0.01 < *P* < 0.05 indicate significance.

The effects of LR and GLN on LPS-induced colonic inflammation were further examined in mice and cells. No difference was found in mouse weight gain among the groups (Fig. S6a). The β-diversity results showed that the structures of the gut microbiota in mice were clearly distinguishable among the groups (Figure 7a, *P* < 0.001). The combination of LR and GLN (MIX) alleviated the LPS-induced decrease in the abundance of *Lactobacillus reuteri* (Figure 7b, LDA > 3.0). MIX restored the morphology of the injured colon and increased the gut permeability caused by LPS (Figure 7c, Fig. S6b, *P* < 0.05). MIX treatment alleviated the LPS-induced decrease in ZO-1, Occludin, and Claudin 1 protein levels (Figure 7d, *P* < 0.05). MIX restored the LPS-induced increase in IL-6 and TNF-α concentrations and the decrease in IL-10 and TGF-β concentrations (Figure 7e, *P* < 0.05). The effects of MIX were better than those of either treatment alone.

The optimal treatment concentrations of LR and GLN were investigated using Caco-2 and 3D4/2 cells in cell viability and cytotoxicity assays (Fig. S6c, d). LR and GLN treatment diminished the LPS-induced decrease in the survival rate of 3D4/2 cells (Fig. S6e, *P* < 0.05). Compared with the CON group, LPS treatment reduced the expression of Arg1 in 3D4/2 cells and increased the expression of iNOS. LR and GLN ameliorated this phenomenon (Fig. S6f, *P* < 0.05).

**Figure 8.**
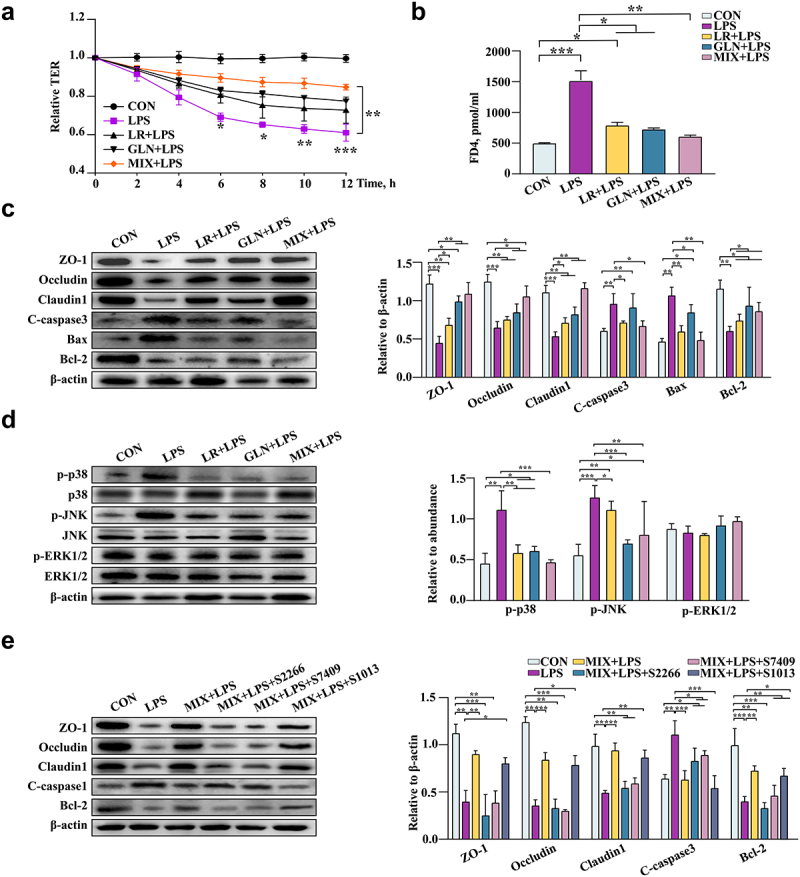
Validation of the effects of LR and GLN on LPS-induced inflammatory injury in cells (n = 3). (a) Changes in the relative TER values of Caco-2 cells for the completely differentiated monolayer; (b) FD4 content in lower compartments of Transwell plates. (c) Tight junction and apoptosis-related protein expression in Caco-2 cells. (d) p38, JNK and ERK1/2 phosphorylation in Caco-2 cells. (e) The tight junction and apoptosis-related protein expression when Caco-2 cells were treated with specific activators of p38, JNK and ERK1/2. ***: *P* < 0.001, **: 0.001 < *P* < 0.01, *: 0.01 < *P* < 0.05 indicate significance.

LR and GLN treatments alleviated the significant LPS-induced decrease in the relative TER values of Caco-2 cells, and the relative TER values of MIX and LPS treatment had the largest difference at 12 h (Figure 8a, *P* < 0.05). LR and GLN significantly inhibited the increase in FD4 content caused by LPS (Figure 8b, *P* < 0.05). Furthermore, LR and GLN alleviated the LPS-induced decrease in the viability of Caco-2 cells (Fig. S6g, *P* < 0.05). LR and GLN restored the LPS-induced decrease in ZO-1, Occludin, Claudin 1 and Bcl-2 expression and increased the expression of cleaved caspase 3 and Bax (Figure 8c, *P* < 0.05). Meanwhile, LR and GLN mitigated the LPS-induced increase in the phosphorylation of p38 MAPK and JNK proteins (Figure 8d, *P* < 0.05). Additionally, the activators of p38 (S2266), JNK (S7409), and ERK1/2 (S1013) were used to explore the effects of MIX on the LPS-induced expression of MAPK-related pathway proteins. The results showed that the three activators effectively altered cell viability and activated the respective proteins of the MAPK pathway (Fig. S6h, i, *P* < 0.05). Remarkably, p38 MAPK and JNK activators significantly reduced the suppressive effects of MIX on the LPS-induced decrease in the cell survival rate and tight junction protein expression and the increase in proapoptotic protein expression, while the ERK1/2 activator had no effect (Figure 8e, *P* < 0.05). The effects of MIX on alleviating LPS-induced inflammation were better than those of LR and GLN alone.

## Discussion

The colonization of the gut by microbes in early life is essential for gut development and contributes to short- and long-term health outcomes.^28^ Maternal nutritional states play fundamental roles in offspring gut health programming.^29^ Fermented diets are functional foods that can positively regulate gut health.^30^ However, there is little evidence to explain how the MFD manipulates gut development and health in offspring. Here, MFD-induced milk GLN and gut LR were found to contribute to early-life gut health by improving the establishment of early gut microbiota and attenuating LPS-induced gut inflammation by suppressing the phosphorylation of p38 MAPK and JNK and activation of Caspase-3.

Gut development in offspring is greatly affected by the maternal gut microbiota and breast milk.^31^ There is little information on the effects of diet on the maternal gut microbiota, although it is important during pregnancy and lactation.^32^ Roytio et al. reported that obese mothers who consumed sufficient amounts of fiber during pregnancy showed low-grade inflammation, a reduced abundance of gut *Bacteroidetes*, and a higher degree of gut microbial richness.^33^ Mandal et al. found that low maternal intake of fat-soluble vitamins was related to increased gut microbial diversity and reduced abundance of *Proteobacteria*.^34^ Only one study indicated that the gut microbial community in lactating women was related to macronutrient and micronutrient intake.^35^ In the present study, the MFD reduced the abundance of maternal gut *Enterobacteriaceae* and *Klebsiella*. The low pH and probiotics in the MFD may have prevented pathogenic infection and enhanced gut health. The MFD significantly promoted the abundance of gut *Lactobacillus* and *Succiniclasticum. Lactobacillus* has been widely studied because of its ability to enhance gut barrier function, balance the gut microbiota, modulate the innate immune system, and prevent the colonization of pathogenic bacteria, thereby benefiting host health.^36^ *Succiniclasticum* can utilize succinate and produce propionate, thus providing benefits to gut health.^37^ The results suggest that MFD not only inhibits pathogens but also plays an important role in the modulation of gut commensal microbiota. The results of the analysis of the milk metabolome indicated that several amino acids and organic acids were significantly upregulated by MFD. Additionally, D-glutamine and D-glutamate metabolism in the milk metabolome was enhanced. Milk metabolites can promote microbial-dependent growth in infants.^38^ Wu et al. reported that breast milk from pathological mothers could impair the growth of newborns.^32^ Thus, the impact of the MFD on gut health and growth performance in offspring was attributed to the manipulation of maternal nutrition as a consequence of the comprehensive effects of the vertical transmission of microbes and milk metabolites. Notably, the correlation analysis revealed that small peptides and the pH of the MFD dramatically shaped the gut microbiota of sows.

Studies have indicated that maternal diets during pregnancy can affect gut health and the risk of pathogen infection in offspring in early and late life.^39–42^ In this study, the effects of the MFD on the longitudinal and horizontal assembly of the gut microbiota in offspring during lactation were investigated. The MFD significantly improved the diversity and abundance of gut *Firmicutes* in offspring on day 28. The results revealed that the effects of the MFD on the development of the gut microbiota depended on time. Interestingly, the MFD accelerated the maturation of the gut microbiota in offspring. Additionally, the microbiota on day 14 had the highest number of correlations and level of abundance, indicating that they may have undergone remarkable changes and play an important role in the development of the gut microbiota in offspring.^41^ Furthermore, the number of gut microbial interaction edges decreased as the offspring aged, demonstrating that the gut microbiota shifted from the initial significant changes to a steady state during lactation. The structural and functional dynamics of the gut microbiome in offspring can be used as an indicator of growth and health.^43^ A mature gut microbiome in suckling offspring is beneficial to host growth and development and prepares animals for weaning.^26^ Therefore, the MFD may improve gut development and health in offspring by promoting the maturation of the gut microbiome. Horizontally, the MFD increased the abundance of gut *Lactobacillus* and *Prevotella* as well as amino acid–related enzyme function and glucose metabolism in piglets on day 28. One human study investigated the associations between maternal diet and gut microbiota in breastfeeding infants.^44^ Babakobi et al. found no correlations between maternal diet and the gut microbiome in offspring due to the difficult specific assessment of maternal dietary consumption during lactation. Two studies reported correlations between maternal fiber consumption and the gut microbiome during pregnancy and lactation in pigs. A decreased abundance of gut *Enterococcus* and an increased abundance of Clostridiaceae were found in piglets from sows fed a fiber-rich diet.^45,46^ The different results may be attributed to the differences in diet type and intake volume, with proper supplementation volumes having the potential to enhance the effects of the maternal diets. In addition, the fermenting strains used to produce the MFD could have affected the results.^47^ To take advantage of their combined probiotic properties, *Bacillus subtilis* and *Enterococcus faecalis* were used as inoculating microbes in the present study, which not only improved the nutritional value of the MFD but also promoted the development of the gut microbiota in offspring by maternal intervention.

Piglets were challenged with LPS to examine the effects of the MFD on the resistance of offspring to colonic inflammation. MFD significantly diminished LPS-induced morphological injury to colonic epithelial cells. The gut epithelial barrier consists of epithelial cells and intercellular junction proteins that play important roles in preventing inflammation and infection.^48^ An increase in the abundance of beneficial microbes in the gut can improve the integrity of the intestinal barrier.^49^ Cheng et al. ^50^ transplanted the fecal microbiota of healthy Jinhua pigs into K88-infected piglets, which were found to have improved intestinal morphology, reduced intestinal permeability and enhanced expression of mucin and mucosal tight junction proteins as a result of the transplant. The MFD improved the establishment of the gut microbiota in piglets and increased the abundance of *Lactobacillus*. These results demonstrated that the MFD benefited the gut microbiota in offspring to mitigate the LPS-induced impairment of colonic physical barriers. Cytokines are involved in the regulation of gut homeostasis and immune function.^51^ In the present study, the MFD restored the cytokine disorder in offspring. Our previous study reported that the serum levels of anti-inflammatory cytokines in sows were enhanced by the MFD.^52^ Maternal immune factors can be transmitted vertically to offspring.^28,53^ Liu et al.^54^ demonstrated that the concentration of IL-10 in maternal serum and feces increased, those of IL-6 and TNF-α decreased, and that of IL-6 in serum of offspring also decreased by adding different dietary supplements to feed sows. A study has also shown that the suppression of colitis was mainly due to the decrease in inflammatory cytokine levels and the increase in anti-inflammatory cytokine levels.^55^ Thus, MFD may ameliorate LPS-induced colonic cytokine disturbances in offspring by improving the immune status of sows. Macrophages comprise proinflammatory macrophages (M1 type) and anti-inflammatory macrophages (M2 type), which play vital roles in the inflammatory response.^56^ MFD significantly alleviated LPS-induced proinflammatory colonic macrophages in offspring, which may be one of the anti-inflammatory mechanisms. Apoptosis is a type of programmed cell death that involves the activation of caspases, which is often accompanied by impairment of the gut barrier.^57^ Therefore, the MFD can diminish apoptosis in the colon of LPS-challenged piglets and promote epithelial barrier function.

FMT is a critical technique for research into the functions of gut microbiota.^58^ To further investigate whether the MFD affected early-life gut health by regulating the maternal gut microbiota, sow fecal microbiota was transplanted to broad-spectrum antibiotic-treated mouse dams. FMT from the MFD notably alleviated the LPS-induced decrease in gut *Lactobacillus* in both mouse dams and pups. Accordingly, FMT from the MFD significantly mitigated LPS-induced colonic inflammation in mouse dams and pups. Inconsistent with the results of the piglets, there was no significant difference in the body weight of pups and an increasing trend in weight gain (*P* = 0.067). The difference in the body weight of the offspring occurred during approximately 8 weeks of maternal prebiotic supplementation in mice.^59^ Thus, the lack of an obvious body weight increase in pups in the present study might be due to insufficient maternal treatment time. Additionally, the different gene types and physiologies between mice and pigs may have caused inconsistencies in the results. Overall, these results are consistent with those of pigs, verifying that the MFD might improve the gut health of offspring by positively modulating the maternal gut microbiota.

Notably, the effects of the MFD were better than those of the PROB in both the maternal and offspring results. This might have been attributed to the probiotics in the MFD, which could positively shape the gut microbiota. At the same time, the MFD contains bioactive compounds, such as hydrolyzed small peptides, which could enhance physical barrier function, activate host immune cells, and improve gut functions. In addition, microbial metabolites in the MFD, such as organic acids, enzymes, and antimicrobial peptides, also exist and exert their properties.^12^

To identify the main maternal components that affect gut health in offspring, correlation analysis and absolute quantification were conducted. The results of correlation analysis indicated that maternal gut LR and milk GLN promoted the abundance of offspring gut LR, thereby improving gut health and animal growth. In addition, the relative and absolute abundance of LR and milk GLN were significantly enriched in the MFD group, indicating that MFD might increase the early-life abundance of LR in the gut by improving maternal gut LR and milk GLN. Furthermore, the *in vitro* fermentation assay with maternal fecal microbiota revealed that the MFD promoted LR proliferation. Therefore, LR and GLN were two of the main maternal effectors that affected gut health in offspring. Additionally, the explanatory analysis indicated that the main contribution to the gut microbiota in offspring was the combined effects of the maternal gut microbiota and milk. Our previous study reported that the content of glutamate increased after diet fermentation.^60^ Thus, the enriched glutamate in the MFD and enhanced milk glutamine metabolism might have contributed to the improved milk GLN. Additionally, the MFD could be easily digested, providing probiotics and their metabolites that might increase the amount of GLN in milk. The underlying mechanism of the increase in the abundance of the offspring gut LR might have been attributed to the transfer of the MFD-improved maternal gut LR through the entero-mammary axis.^7^ LR and GLN alleviated LPS-induced gut inflammatory disorders by inhibiting p38 MAPK/JNK phosphorylation. The MAPK pathway plays crucial roles in cell physiology and the immune response.^61^ MFD mitigated the LPS-induced increased phosphorylation of p38 MAPK and JNK in the colon during early life, which might have contributed to the reduced colonic inflammation. Ulcerative colitis can be ameliorated by inhibiting MAPK activation and mediating gut barrier integrity.^62^ Therefore, it is speculated that MFD might have alleviated the LPS-induced increase in the phosphorylation of p38 MAPK and JNK, thus reducing colonic inflammation in offspring. LR can secrete reuterin and lactic acid to improve gut health and enhance immunity.^63^ Although GLN is not generally considered an essential nutrient, it can improve gut mucosa and barrier functions and affect the expression of amino acid receptors and transporters as well as the immune function of the gut.^64^ Therefore, the beneficial effects of gut LR and milk GLN on LPS-induced inflammation *in vivo* and *in vitro* were further revealed. Interestingly, LR and GLN together had the best effect, further implying that the combination of maternal milk and gut microbiota plays a major role in regulating the gut health of offspring.

## Conclusions

The results revealed that an MFD benefited the performance of sows and piglets, the maternal gut microbiota, and the metabolite content of the milk from the sows. Meanwhile, the MFD accelerated the maturation of the neonatal gut microbiota, promoted the abundance of gut LR, and enhanced microbial metabolic functions. In addition, the MFD regulated the neonatal gut microbiota and epithelial homeostasis through the maternal gut microbiota. Milk GLN and gut LR were two MFD-caused maternal effectors that regulated gut microbiota development and alleviating LPS-induced colonic inflammation in offspring by suppressing the phosphorylation of p38 MAPK and JNK and activation of Caspase-3. These results expand our knowledge of preemptive strategies for neonatal intestinal inflammation that target both maternal factors and the early-life gut microbiota; furthermore, these results indicate that an MFD may be valuable in regulating gut health in offspring.

## Supplementary Material

Supplemental Material

## Data Availability

The datasets supporting the conclusions of this article are available in the NCBI Sequence Read Archive (SRA) repository under accession number PRJNA552,228, 765,737, 765,800 and 765,829.

## Abbreviations

**MFD**: Maternal fermented diet

**LR**: *Lactobacillus reuteri*

**GLN**: L-glutamine

**LPS**: Lipopolysaccharide

**MAPK**: Mitogen-activated protein kinase

**JNK**: c-Jun N-terminal kinases

**ERK**: Extracellular signal regulated kinase

**FMT**: Faecal microbiota transplantation

**PROB**: Probiotics

**TUNEL**: Terminal deoxynucleotidyl transferase-mediated dUTP nick end labeling

**PCoA**: Principal co-ordinates analysis

**SEM**: Scanning electron microscope

**SPF**: Specific pathogen-free

**TEM**: Transmission electron microscopy

**PBS**: Phosphate buffer saline
