# Supplementary material for: Maternal consumption of a fermented diet protects offspring against intestinal inflammation by regulating the gut microbiota
Source: Gut Microbes. 2022 May 4;14(1):2057779. doi: 10.1080/19490976.2022.2057779 (PMC9090288; doi:10.1080/19490976.2022.2057779)
Supplement: Supplemental Material [file KGMI_A_2057779_SM2109.docx]

**Figure S1
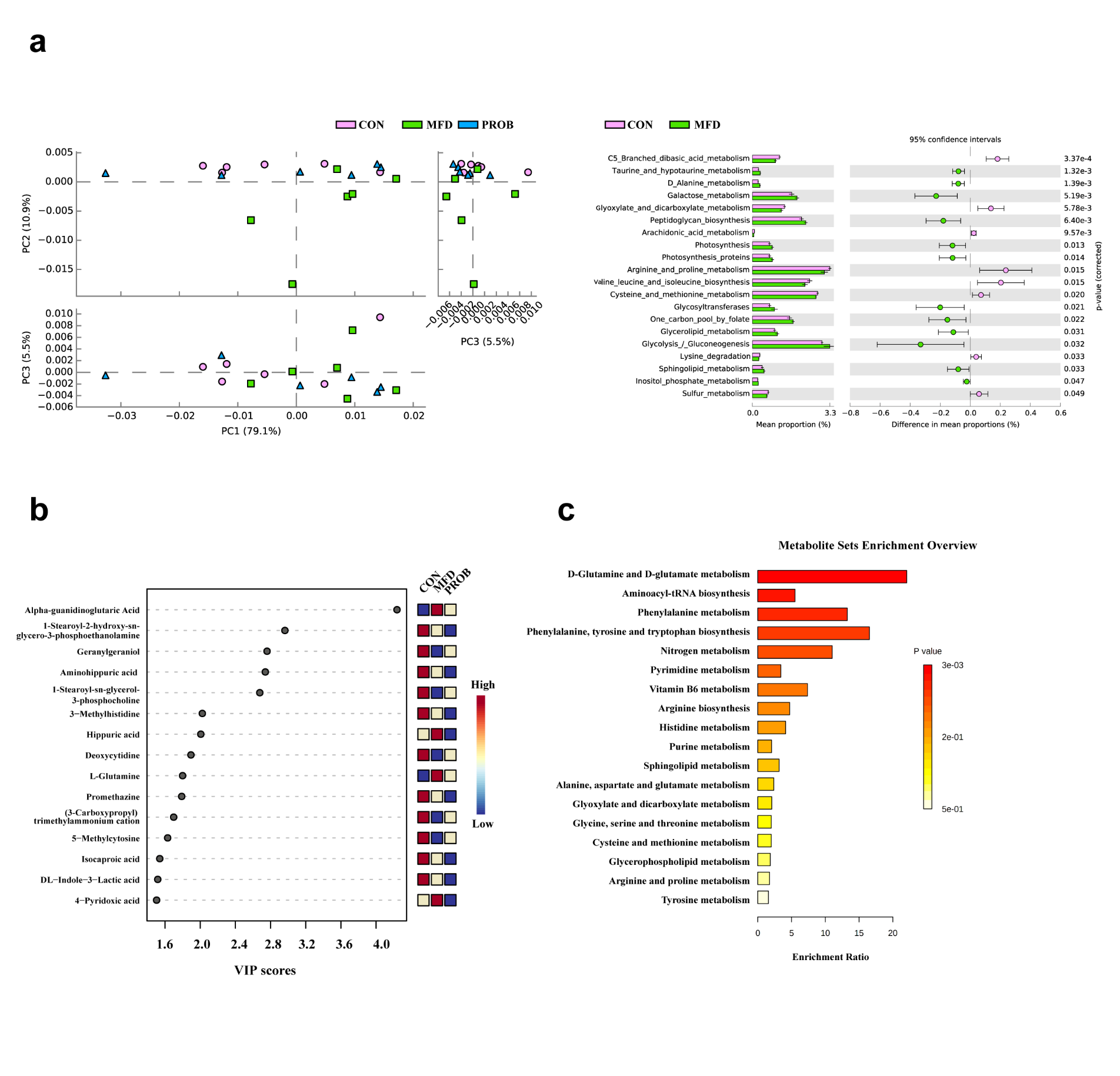
**

**Figure S2
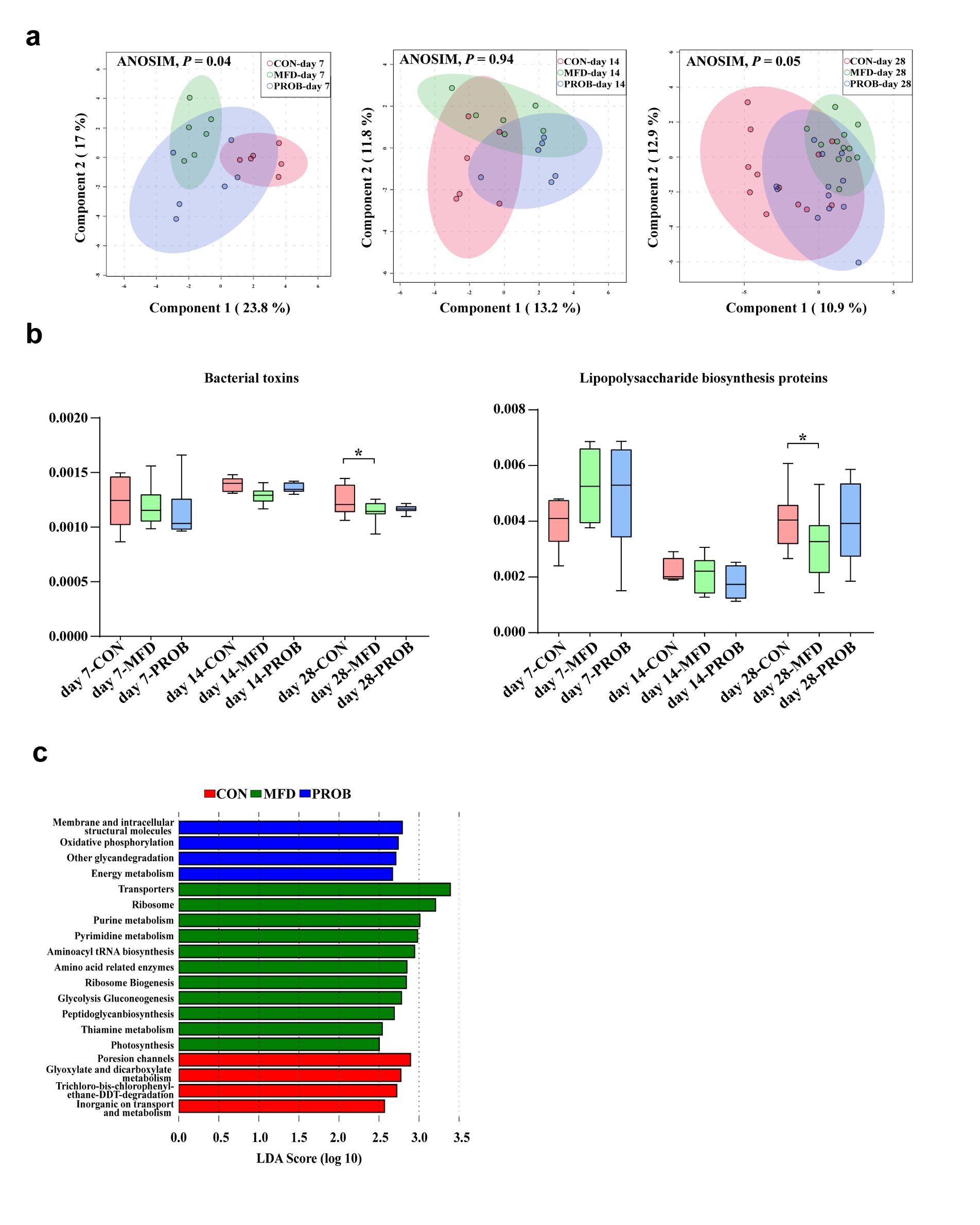
**

**Figure S3**

**
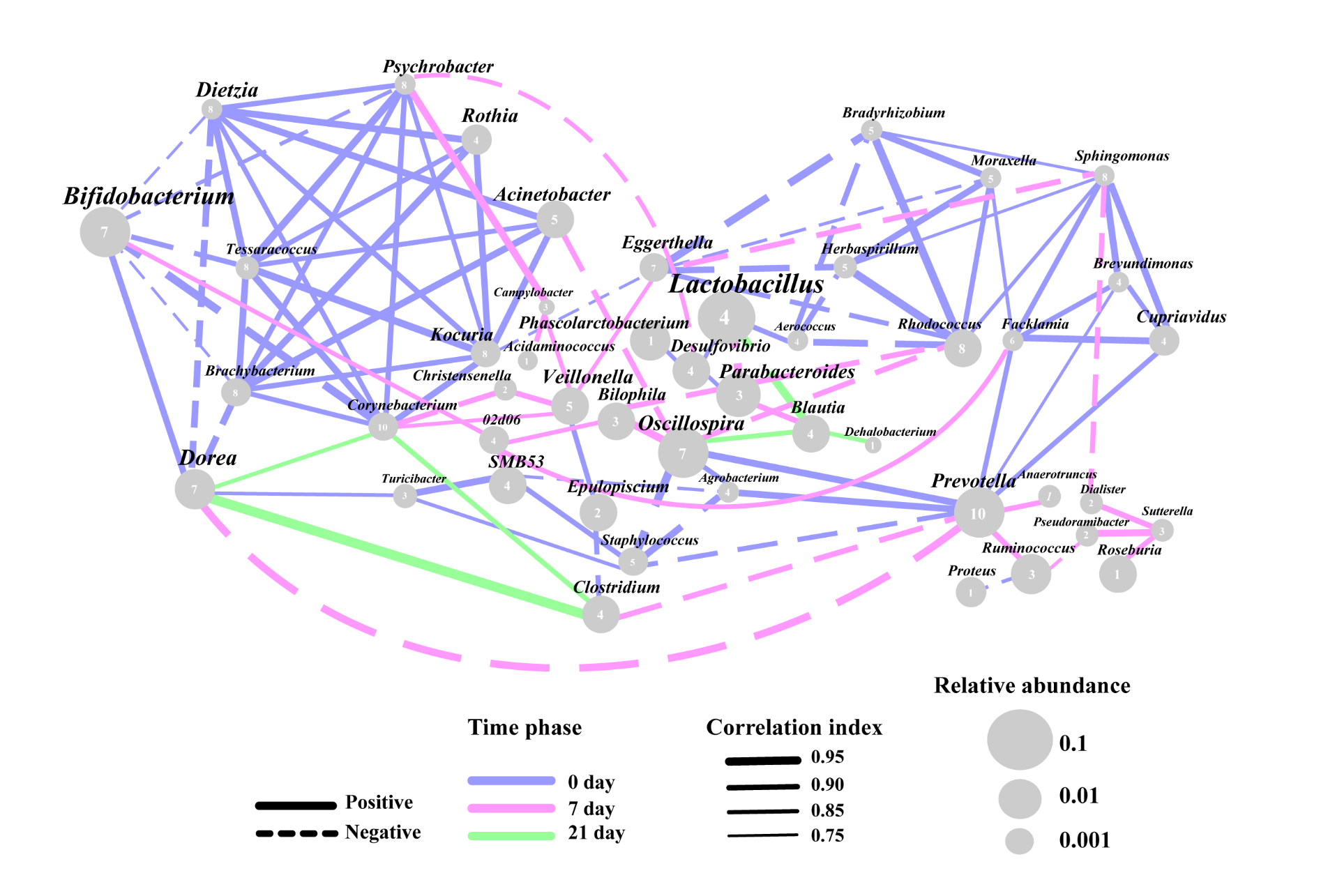
Figure S4**

**
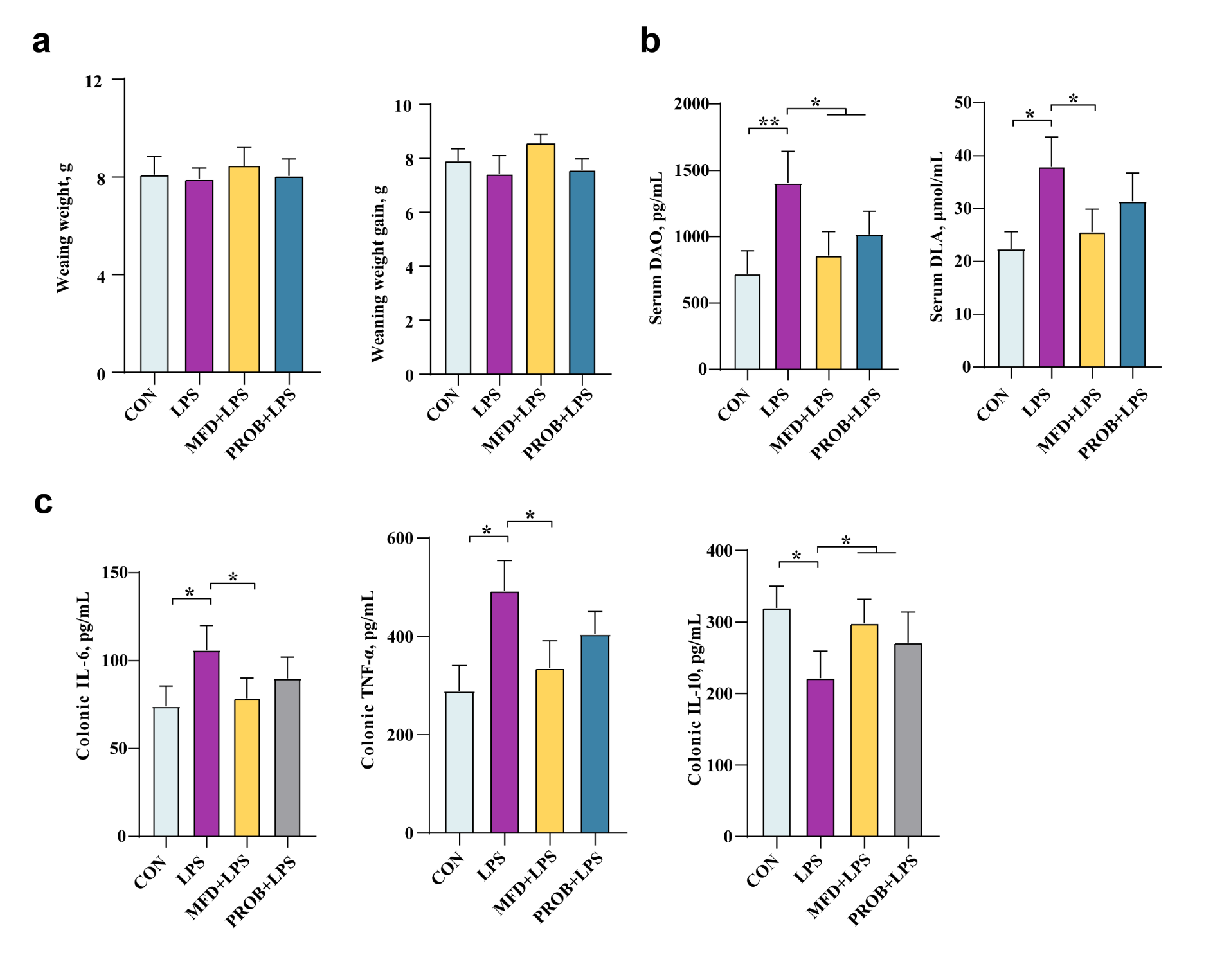
**

**Figure S5**

**
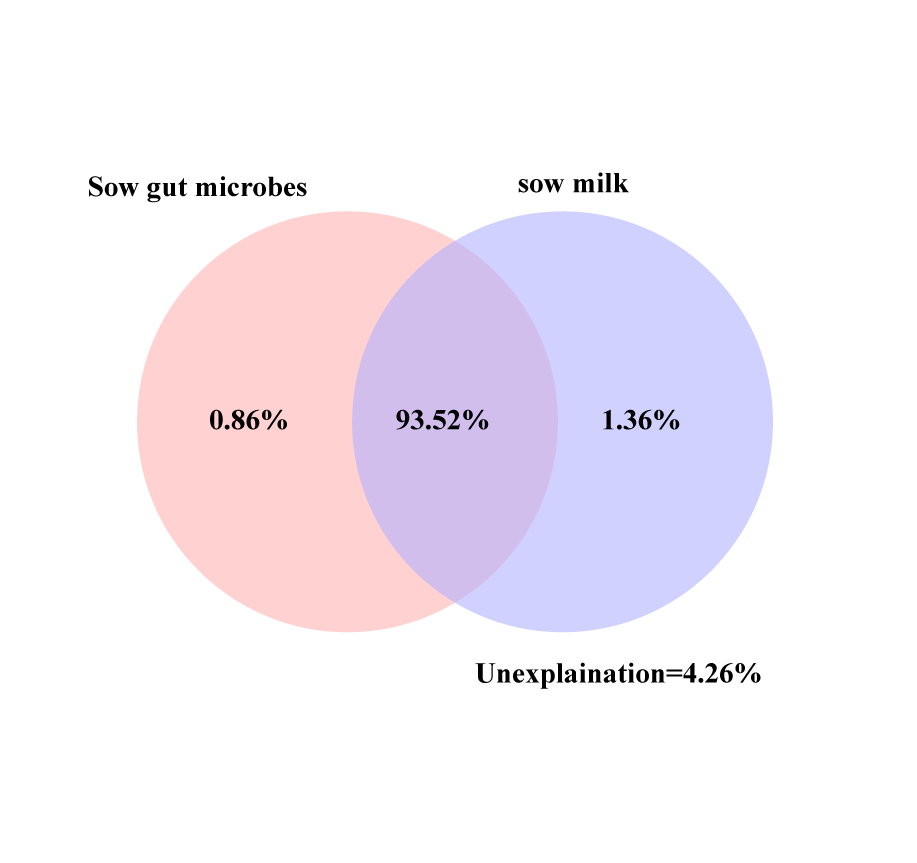
**

**Figure S6**


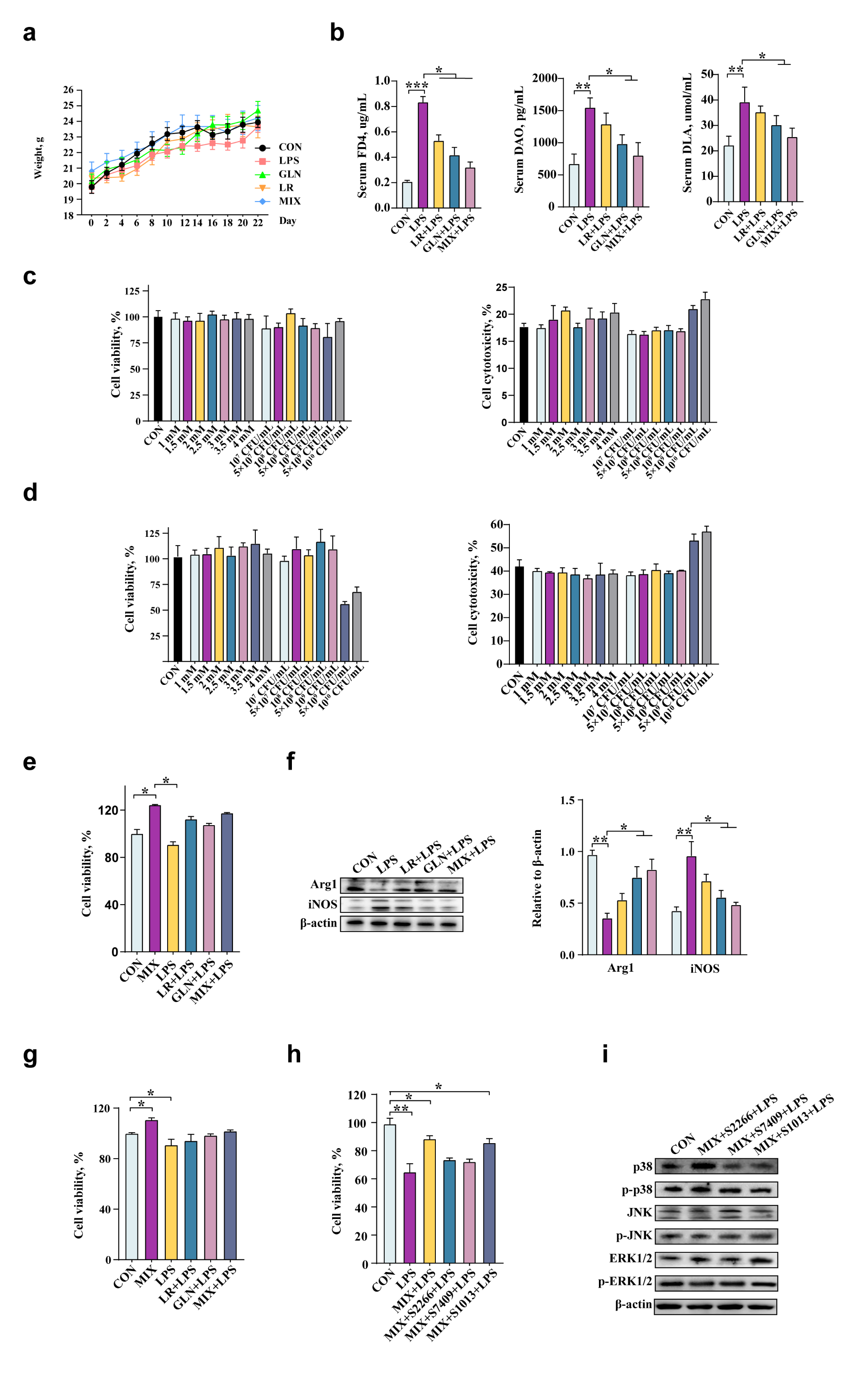


**Table S1.** Nutrient composition of the MFD (as-fed basis, n=3)^1^

| Item | Maternal unfermented diet | | Maternal fermented diet | SEM | *P*-value |
| --- | --- | --- | --- | --- | --- |
| Dry matter, % | | 88.89 | 90.04 | 1.25 | 0.57 |
| Crude protein, % | | 27.16 | 29.54 | 0.21 | 0.02 |
| Small peptides, % | | 3.35 | 12.41 | 2.45 | 0.00 |
| NDF, % | | 15.42 | 9.03 | 1.02 | 0.01 |
| ADF, % | | 6.87 | 5.31 | 0.76 | 0.03 |
| Amylose, % | | 5.86 | 2.32 | 0.49 | 0.04 |
| Ether extract,^5^ % | | 3.67 | 3.37 | 0.18 | 0.25 |
| Ash, % | | 3.89 | 4.17 | 0.21 | 0.44 |
| Ca, % | | 0.17 | 0.19 | 0.02 | 0.63 |
| Total phosphorus, % | | 0.49 | 0.54 | 0.04 | 0.54 |
| pH | | 6.34 | 4.67 | 0.39 | 0.03 |
| Lactic acid, mmol/kg | | – | 154.73 | - | - |
| Live *Bacillus subtilis*, cfu/g | | – | 5.7 × 10^8^ | - | - |
| Live *Enterococcus faecium*, cfu/g | | – | 5.9× 10^8^ | - | - |

^1^ The diet consists of 40% corn, 40% soybean meal, and 20% yellow wine lees.

**Table S2.** Differential diet information of MFD and differential gut microbiota of sows (n=6)

| **Item** | | | **CON1** | **CON2** | **CON3** | **CON4** | **CON5** | **CON6** | **MFD1** | **MFD2** | **MFD3** | **MFD4** | **MFD5** | **MFD6** | **PROB1** | **PROB2** | **PROB3** | **PROB4** | **PROB5** | **PROB6** |
| --- | --- | --- | --- | --- | --- | --- | --- | --- | --- | --- | --- | --- | --- | --- | --- | --- | --- | --- | --- | --- |
| **Diet** | **g_*Bacillus*, %** | **0.0030** | | **0.0015** | **0.0035** | **0.0018** | **0.0035** | **0.0018** | **0.5054** | **0.4865** | **0.4470** | **0.4002** | **0.4470** | **0.4002** | **0.5054** | **0.4865** | **0.4470** | **0.4002** | **0.4470** | **0.4002** |
|  | **g_*Enterococcus*, %** | **0.0003** | | **0.0002** | **0.0001** | **0.0002** | **0.0001** | **0.0002** | **0.4832** | **0.4812** | **0.5252** | **0.5841** | **0.5252** | **0.5841** | **0.4832** | **0.4812** | **0.5252** | **0.5841** | **0.5252** | **0.5841** |
|  | **g_*Pseudomonas*, %** | **0.0134** | | **0.0113** | **0.0142** | **0.0113** | **0.0142** | **0.0113** | **0.0034** | **0.0288** | **0.0220** | **0.0121** | **0.0220** | **0.0121** | **0.0134** | **0.0113** | **0.0142** | **0.0113** | **0.0142** | **0.0113** |
|  | **g_*Staphylococcus*, %** | **0.0002** | | **0.0000** | **0.0001** | **0.0000** | **0.0001** | **0.0000** | **0.0002** | **0.0001** | **0.0002** | **0.0002** | **0.0002** | **0.0002** | **0.0002** | **0.0000** | **0.0001** | **0.0000** | **0.0001** | **0.0000** |
|  | **g_*Pediococcus*, %** | **0.0000** | | **0.0000** | **0.0000** | **0.0000** | **0.0000** | **0.0000** | **0.0030** | **0.0002** | **0.0005** | **0.0001** | **0.0005** | **0.0001** | **0.0000** | **0.0000** | **0.0000** | **0.0000** | **0.0000** | **0.0000** |
|  | **g_*Facklamia*, %** | **0.0000** | | **0.0000** | **0.0000** | **0.0000** | **0.0000** | **0.0000** | **0.0002** | **0.0004** | **0.0016** | **0.0003** | **0.0016** | **0.0003** | **0.0000** | **0.0000** | **0.0000** | **0.0000** | **0.0000** | **0.0000** |
|  | **pH** | **6.10** | | **6.12** | **6.05** | **5.89** | **6.11** | **5.98** | **5.42** | **5.65** | **5.56** | **5.63** | **5.73** | **5.49** | **5.78** | **6.12** | **6.04** | **5.89** | **5.93** | **6.04** |
|  | **Small peptides, %** | **3.54** | | **4.45** | **4.04** | **3.89** | **2.78** | **4** | **7.34** | **6.34** | **7.32** | **7.54** | **6.98** | **7.03** | **3.67** | **4.03** | **4.14** | **3.95** | **3.14** | **4.34** |
|  | **Live probiotics, log CFU/g CFU/g** | **0.00** | | **0.00** | **0.00** | **0.00** | **0.00** | **0.00** | **4.51** | **4.14** | **3.95** | **5.06** | **4.72** | **4.6** | **4.35** | **4.68** | **4.32** | **5.03** | **3.67** | **4.33** |
| **Sow’s gut microbiota** | **f_*Clostridiaceae*, %** | **0.0172** | | **0.0912** | **0.0306** | **0.0317** | **0.0869** | **0.0157** | **0.0035** | **0.0123** | **0.0080** | **0.0070** | **0.0079** | **0.0059** | **0.0296** | **0.0516** | **0.0136** | **0.0094** | **0.0170** | **0.0128** |
|  | **f_*Enterobacteriaceae*, %** | **0.0576** | | **0.0419** | **0.0110** | **0.0041** | **0.0605** | **0.0269** | **0.0000** | **0.0146** | **0.0009** | **0.0006** | **0.0056** | **0.0107** | **0.0010** | **0.0025** | **0.0063** | **0.0002** | **0.0019** | **0.0038** |
|  | **g_*Turicibacter*, %** | **0.0024** | | **0.0094** | **0.0097** | **0.0066** | **0.0248** | **0.0216** | **0.0002** | **0.0028** | **0.0026** | **0.0004** | **0.0022** | **0.0006** | **0.0086** | **0.0071** | **0.0050** | **0.0006** | **0.0033** | **0.0010** |
|  | **g_*Klebsiella*, %** | **0.0000** | | **0.0001** | **0.0001** | **0.0000** | **0.0000** | **0.0003** | **0.0000** | **0.0000** | **0.0000** | **0.0000** | **0.0000** | **0.0000** | **0.0000** | **0.0000** | **0.0000** | **0.0000** | **0.0000** | **0.0000** |
|  | **g_*Clostridium*, %** | **0.0014** | | **0.0084** | **0.0018** | **0.0028** | **0.0120** | **0.0018** | **0.0012** | **0.0009** | **0.0004** | **0.0009** | **0.0015** | **0.0003** | **0.0032** | **0.0119** | **0.0028** | **0.0011** | **0.0070** | **0.0005** |
|  | **g_*SMB53*, %** | **0.0010** | | **0.0020** | **0.0027** | **0.0019** | **0.0020** | **0.0022** | **0.0001** | **0.0006** | **0.0003** | **0.0005** | **0.0003** | **0.0002** | **0.0030** | **0.0023** | **0.0008** | **0.0000** | **0.0020** | **0.0017** |
|  | **f_*Peptostreptococcaceae*, %** | **0.0011** | | **0.0020** | **0.0028** | **0.0038** | **0.0080** | **0.0027** | **0.0008** | **0.0012** | **0.0013** | **0.0013** | **0.0010** | **0.0005** | **0.0024** | **0.0034** | **0.0009** | **0.0005** | **0.0025** | **0.0015** |
|  | **g_*Lactobacillus*, %** | **0.0481** | | **0.0012** | **0.0017** | **0.0002** | **0.0004** | **0.0018** | **0.0691** | **0.1709** | **0.4782** | **0.1223** | **0.0963** | **0.2703** | **0.0018** | **0.0532** | **0.0169** | **0.0348** | **0.0501** | **0.0598** |
|  | **g_*Succiniclasticum*, %** | **0.0000** | | **0.0000** | **0.0000** | **0.0000** | **0.0000** | **0.0000** | **0.0001** | **0.0004** | **0.0006** | **0.0001** | **0.0000** | **0.0004** | **0.0000** | **0.0000** | **0.0000** | **0.0000** | **0.0000** | **0.0000** |
|  | **f_*WCHB1-25*, %** | **0.0001** | | **0.0000** | **0.0000** | **0.0000** | **0.0000** | **0.0000** | **0.0015** | **0.0002** | **0.0001** | **0.0000** | **0.0000** | **0.0002** | **0.0000** | **0.0000** | **0.0000** | **0.0000** | **0.0000** | **0.0000** |
|  | **c_*GKS2-174*, %** | **0.0000** | | **0.0000** | **0.0000** | **0.0000** | **0.0000** | **0.0000** | **0.0006** | **0.0000** | **0.0000** | **0.0000** | **0.0001** | **0.0001** | **0.0000** | **0.0002** | **0.0001** | **0.0001** | **0.0000** | **0.0000** |
|  | **g_*rc4-4*, %** | **0.0000** | | **0.0000** | **0.0000** | **0.0000** | **0.0000** | **0.0001** | **0.0000** | **0.0000** | **0.0000** | **0.0000** | **0.0000** | **0.0000** | **0.0006** | **0.0000** | **0.0001** | **0.0002** | **0.0000** | **0.0001** |
|  | **o_*RF39*, %** | **0.0101** | | **0.0108** | **0.0098** | **0.0045** | **0.0092** | **0.0001** | **0.0080** | **0.0075** | **0.0047** | **0.0051** | **0.0133** | **0.0035** | **0.0342** | **0.0148** | **0.0113** | **0.0107** | **0.0072** | **0.0141** |
|  | **f_*Erysipelotrichaceae*, %** | **0.0039** | | **0.0011** | **0.0045** | **0.0032** | **0.0029** | **0.0010** | **0.0010** | **0.0012** | **0.0012** | **0.0036** | **0.0036** | **0.0020** | **0.0052** | **0.0024** | **0.0038** | **0.0055** | **0.0043** | **0.0039** |
|  | **g_*Mitsuokella*, %** | **0.0001** | | **0.0002** | **0.0000** | **0.0000** | **0.0002** | **0.0001** | **0.0012** | **0.0003** | **0.0001** | **0.0003** | **0.0007** | **0.0003** | **0.0003** | **0.0011** | **0.0004** | **0.0002** | **0.0004** | **0.0007** |
|  | **o_*GMD14H09*, %** | **0.0002** | | **0.0000** | **0.0000** | **0.0000** | **0.0000** | **0.0001** | **0.0012** | **0.0011** | **0.0001** | **0.0001** | **0.0010** | **0.0022** | **0.0032** | **0.0002** | **0.0018** | **0.0008** | **0.0012** | **0.0003** |

**Table S3.** Composition and nutritional value of the experimental diets (as-fed basis, n=3)

| Item | Diet | | |
| --- | --- | --- | --- |
|  | Control | 10% Fermented diet | Probiotics |
| Ingredients | | | |
| Corn | 50 | 50 | 50 |
| Soybean | 10.0 | 7.0 | 10.0 |
| Extruded soybean | 14.0 | 14.0 | 14.0 |
| Alfalfa meal | 3.0 | 3.0 | 3.0 |
| Fish meal | 3.0 | 3.0 | 3.0 |
| Soy oil | – | 3 | – |
| Unfermented substrates | 10 | - | 10 |
| Fermented substrates | – | 10 | – |
| Yeast hydrolysate | 3.8 | 3.8 | 3.8 |
| Citric acid | – | – | – |
| Baking soda | 0.2 | 0.2 | 0.2 |
| Salt | 0.40 | 0.40 | 0.4 |
| Limestone | 0.6 | 0.6 | 0.6 |
| Premix^1^ | 5.0 | 5.0 | 5.0 |
| Total | 100.00 | 100.00 | 100.00 |
| Nutrition composition | | | |
| Gross energy, MJ/kg | 15.23 | 15.66 | 15.32 |
| Dry matter, % | 88.55 | 87.89 | 87.63 |
| Crude protein, % | 17.32 | 17.24 | 17.23 |
| Ether extract, % | 5.05 | 4.95 | 5.13 |
| Ash, % | 6.34 | 6.27 | 6.23 |
| Calcium, % | 0.95 | 0.98 | 1.02 |
| Total phosphorus, % | 0.48 | 0.47 | 0.47 |
| pH | 6.11 | 5.46 | 6.02 |
| Live *Bacillus subtilis* CFU/g | – | 8.2×10^5^ | 8.7×10^5^ |
| Live *Enterococcus faecium*, CFU/g | – | 2.4×10^5^ | 2.5×10^5^ |

^1^Provided quantities of the following vitamins per kilogram of the complete diet: 10,000 IU vitamin A as vitamin A acetate, 1,500 IU vitamin D_3_ as d-activated animal sterol, 50 IU vitamin E as alpha tocopherol acetate, 4.4 mg vitamin K_3_ as menadione dimethylpyrimidinol bisulfite, 3.0 mg thiamin as thiamine mononitrate, 6.0 mg riboflavin, 3.0 mg pyridoxine as pyridoxine hydrochloride, 0.04 mg vitamin B_12_, 23 mg d-pantothenic acid as calcium pantothenate, 36 mg niacin, 0.8 mg folic acid, 0.15 mg biotin, and 186 mg choline as choline chloride. Also provided the following quantities of minerals per kilogram of the complete diet: 50 mg Cu as copper sulfate, 80 mg Fe as ferrous sulfate, 0.30 mg I as potassium iodate, 20 mg Mn as manganese sulfate, 0.2 mg Se as sodium selenite, and 95 mg Zn as zinc sulfate.

**Table S5.** Effects of the MFD and probiotics on the productivity of the sows and piglets (n=20).

| Item | Diet | | | SEM | *P*-value |
| --- | --- | --- | --- | --- | --- |
|  | Control | MFD | PROB |  |  |
| Sows | | | | | |
| ADFI,^1^ kg/d | 5.76^b^ | 6.61^a^ | 5.89^b^ | 0.07 | 0.01 |
| Milk yield, Kg | 8.60^b^ | 9.02^a^ | 8.80^b^ | 0.07 | 0.03 |
| Piglets | | | | | |
| Number at birth, total | 11.67 | 12.33 | 12.00 | 0.31 | 0.71 |
| Number at birth, live | 11.50 | 12.00 | 11.67 | 0.25 | 0.73 |
| Number at weaning | 11.00 | 11.67 | 11.33 | 0.24 | 0.56 |
| Weaning alive rate,^2^ % | 95.22 | 97.33 | 97.77 | 0.99 | 0.79 |
| Wt at birth,^3^ kg | 1.20 | 1.23 | 1.22 | 0.01 | 0.62 |
| Wt at weaning,^4^ kg | 5.12^b^ | 5.56^a^ | 5.34^b^ | 0.06 | 0.05 |
| Wt gain,^5^ kg | 3.92^b^ | 4.33^a^ | 4.12^b^ | 0.06 | 0.04 |
| Diarrhea incidence,^6^ % | 1.48^a^ | 1.12^c^ | 1.24^b^ | 0.04 | 0.00 |

^a,b^Means within a row with different superscripts significantly differ (*P* < 0.05).

^1^ADFI of the sows were recorded from parturition until weaning (21 d).

^2^Weaning alive rate = [litter size at weaning (live) − litter size at birth (live)]/litter size at birth (live).

^3^Piglet weight at birth = litter weight at birth/litter size at birth (live).

^4^Piglet weight at weaning = litter weight at weaning/litter size at weaning (live).

^5^Piglet weight gain = piglet weight at weaning − piglet weight at birth.

^6^Diarrhea incidence = total diarrhea piglets/[litter size at birth (live) × trial days].

All the values contained six repetitions.

**Table S6.** NCBI BLAST results for the most significant OTU sequence of *Lactobacillus* in the gut of sows.

| Description | Total Score | E value | Per. Ident | Accession |
| --- | --- | --- | --- | --- |
| *Lactobacillus reuteri* TD1 | 1319 | 2e-54 | 99.18% | NC_021872.1 |
| *Lactobacillus vaginalis strain* LV515 | 1071 | 2e-54 | 99.18% | NZ_CP045240.1 |
| *Lactobacillus manihotivorans strain* LM010 | 1004 | 2e-49 | 96.72% | NZ_CP045068.1 |
| *Lactobacillus frumenti strain* LF145 | 883 | 2e-54 | 99.18% | NZ_CP044534.1 |
| *Lactobacillus antri* DSM16041 SCAFFOLD1 | 220 | 2e-54 | 99.18% | NZ_GG700732.1 |
| *Lactobacillus panis* DSM 6035 NODE_207 | 220 | 2e-54 | 99.18% | NZ_AZGM01000112.1 |
| *Lactobacillus o*ris F0423 | 220 | 2e-54 | 99.18% | NZ_AFTL01000020.1 |
| *Lactobacillus oris* F0423 | 220 | 2e-54 | 99.18% | NZ_AFTL01000012.1 |
| *Lactobacillus oris* F0423 | 220 | 2e-54 | 99.18% | NZ_AFTL01000008.1 |
| *Lactobacillus oris* F0423 | 220 | 2e-54 | 99.18% | NZ_AFTL01000006.1 |
| *Lactobacillus oris* F0423 | 220 | 2e-54 | 99.18% | NZ_AFTL01000005.1 |
| *Delftia tsuruhatensis strain* MTQ3 | 209 | 3e-51 | 97.54% | NZ_LCZH01000046.1 |
| *Lactobacillus porcinae strain JCM* | 204 | 2e-49 | 96.72% | NZ_RHNS01000032.1 |
| *Lactobacillus pontis strain* | 204 | 2e-49 | 96.72% | NZ_PNFV01000020.1 |
| *Lactobacillus cameliae* DSM 22697=JCM 13995 *strain* DSM 22697 | 204 | 2e-49 | 96.72% | NZ_AYZJ01000044.1 |

**Table S7.** NCBI BLAST results for the most significant OTU sequence of *Lactobacillus* in the gut of piglets.

| Description | Total Score | E value | Per. Ident | Accession |
| --- | --- | --- | --- | --- |
| *Lactobacillus reuteri* TD1 | 2760 | 2e-126 | 99.22% | NC_021872.1 |
| *Lactobacillus vaginalis strain* LV515 | 2321 | 4e-128 | 99.61% | NZ_CP045240.1 |
| *Lactobacillus frumenti strain* LF145 | 1843 | 2e-126 | 99.22% | NZ_CP044534.1 |
| *Lactobacillus antri* DSM 16041 | 460 | 2e-126 | 99.22% | NZ_GG700732.1 |
| *Lactobacillus panis* DSM 6035NODE_207 | 460 | 2e-126 | 99.22% | NZ_AZGM01000112.1 |
| *Lactobacillus oris* F0423 | 460 | 2e-126 | 99.22% | NZ_AFTL01000020.1 |
| *Lactobacillus oris* F0423 | 460 | 2e-126 | 99.22% | NZ_AFTL01000012.1 |
| *Lactobacillus oris* F0423 | 460 | 2e-126 | 99.22% | NZ_AFTL01000008.1 |
| *Lactobacillus oris* F0423 | 460 | 2e-126 | 99.22% | NZ_AFTL01000006.1 |
| *Lactobacillus oris* F0423 | 460 | 2e-126 | 99.22% | NZ_AFTL01000005.1 |
| *Lactobacillus pontis strain* | 444 | 2e-121 | 98.05% | NZ_PNFV01000020.1 |
| *Lactobacillus raoultii strain Marseille-*P4006 | 433 | 4e-118 | 97.27% | NZ_LS422986.1 |
| *Lactobacillus parafarraginis strain* FAM 1079 | 427 | 2e-116 | 96.88% | NZ_VBSX01000010.1 |
| *Lactobacillus diolivorans* DSM 14421 | 427 | 2e-116 | 96.88% | NZ_AZEY01000081.1 |
